# Supplementary material for: Clonal somatic copy number altered driver events inform drug sensitivity in high-grade serous ovarian cancer
Source: Nat Commun. 2022 Oct 26;13:6360. doi: 10.1038/s41467-022-33870-0 (PMC9606297; doi:10.1038/s41467-022-33870-0)
Supplement: Supplementary file 1 — Supplementary Information [file 41467_2022_33870_MOESM1_ESM.pdf]

Supplementary Figure S1

a

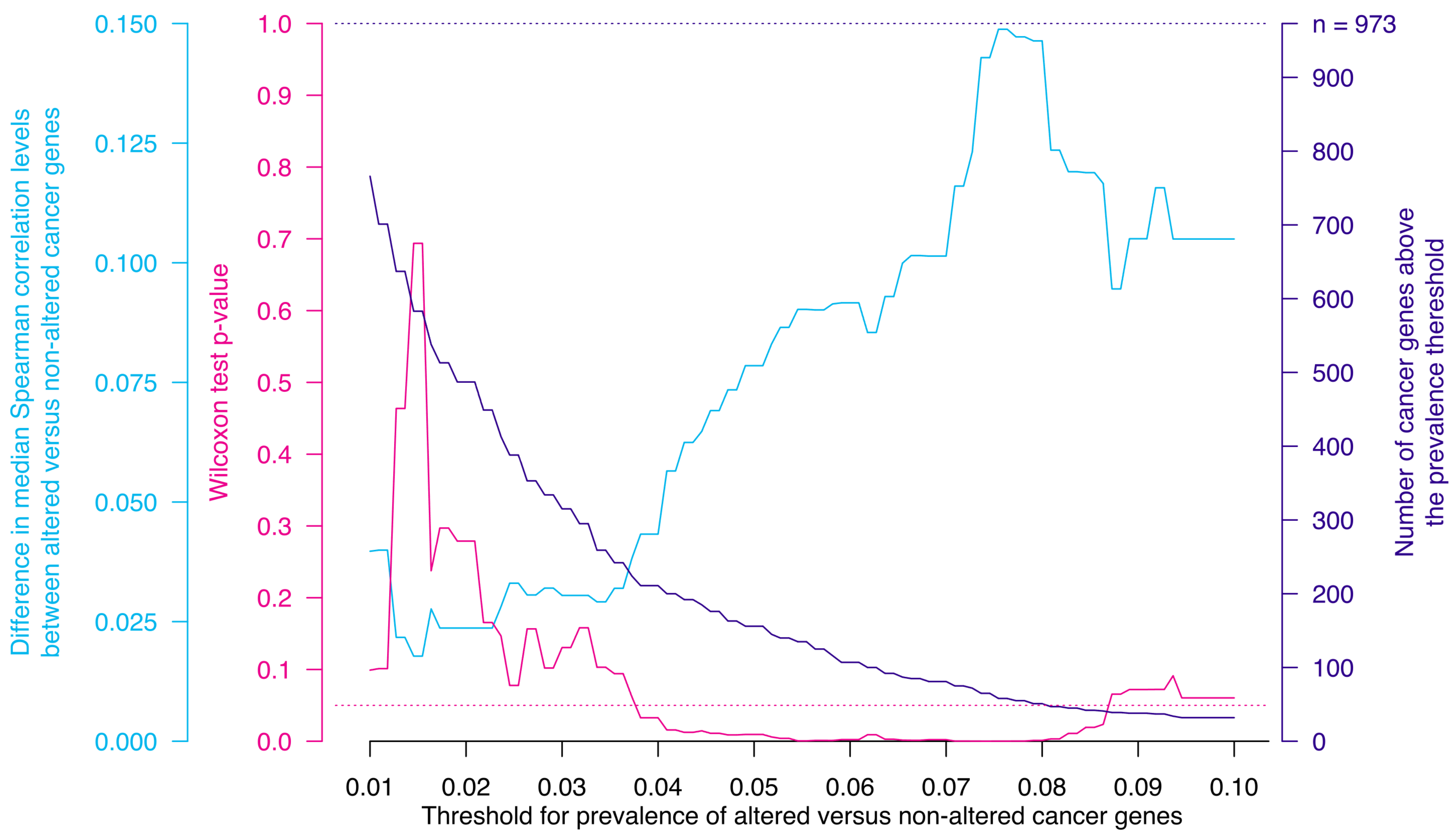

b

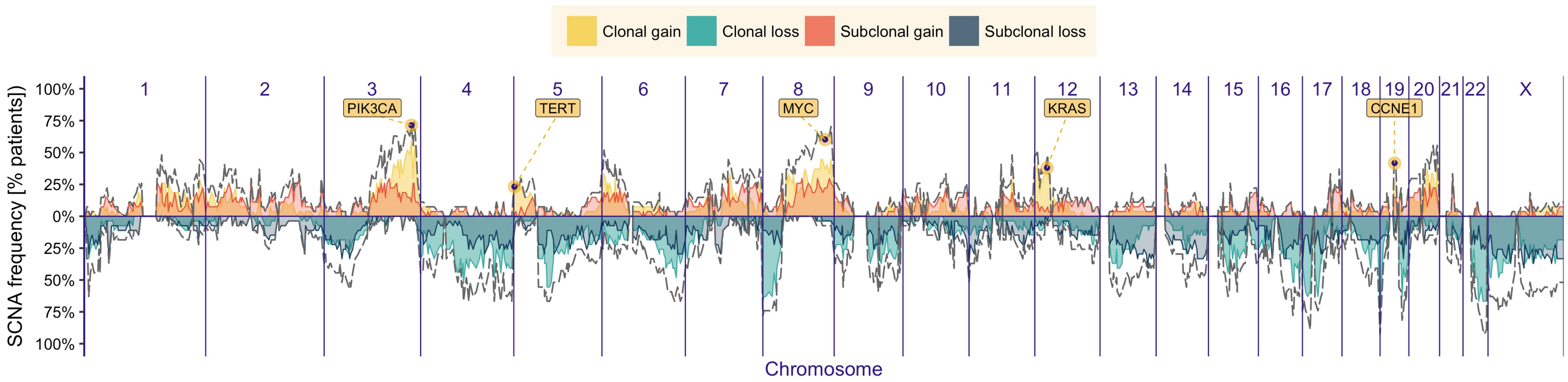

a. Plot showing, for different threshold values defining altered versus non-altered genes (x-axis), the number of cancer genes above the threshold (purple line), the difference of median of Spearman's correlation scores between gene expression and respective chromosomal copy number for each gene (light blue line) and the p-values of the two-sided Spearman's test of association between paired samples when comparing altered cancer genes with remaining cancer genes (pink line) **b.** Genomic distribution of the frequency of clonal and subclonal somatic copy number alterations across 127 regions of both primary tumours and metastases from 30 HGSOC patients.

Supplementary Figure S2

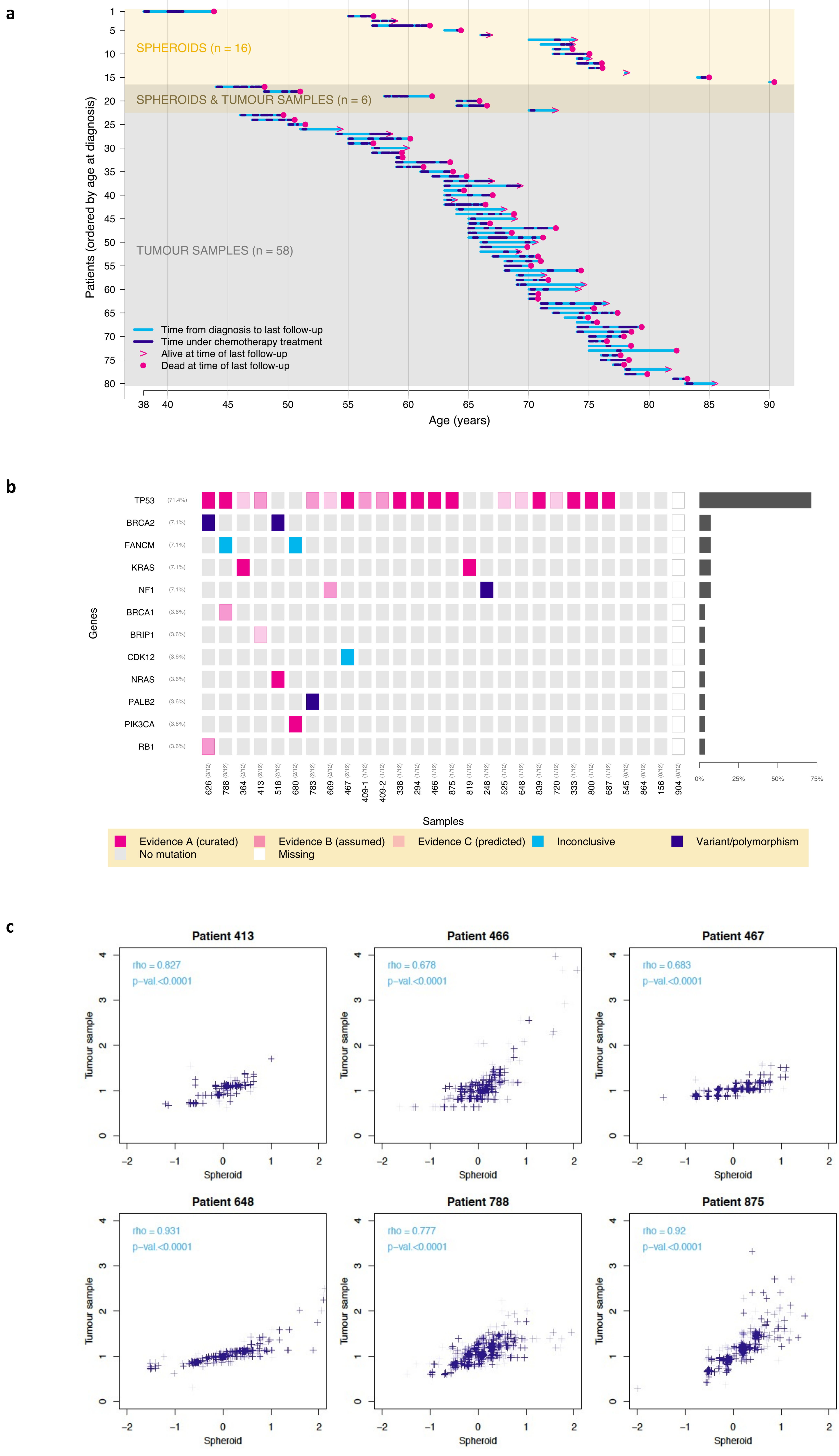

a. Timeline with characterisation of the OVO4 clinical cohort used in this study and time of follow-up for each patient with information related to patient status at last follow-up, chemotherapy treatment period(s) and cancer type. Please see Supplementary Figures S3a-u for more clinical details on the cohort of spheroid samples. **b.** Matrix showing the gene variants (mutations and polymorphisms) identified in our cohort, distributed by functionality and level of evidence (after curation using the MTBP clinical decision support system. **c.** Spearman's correlation coefficient and two-sided test p-value (inference without multiplicity correction) between relative copy number for each individual genomic segment in matched spheroid and tumour samples.

Supplementary Figure S3

Supplementary Figures S3a-u (figures in the following pages)

Summary of clinical details, genomic profiles and *in vitro* response to several targeted drugs for each individual HGSOC spheroid sample. In the timelines on the top left quadrant, we have marked relevant time points, including S: day of surgery, C: date for sample collection, D: death; CT is represented as blue (complete response), green (partial response), yellow (stable disease) and red (progressive disease). Chemotherapy treatment periods and drug combinations are available in the lower part of the plot. In the top right quadrant, we present the genomic profiles for each spheroid sample. In the x-axis, the genomic segments from each chromosome are in order and, in the y-axis, the relative copy number is presented in log 2 scale. Plots showing individual drug response in spheroids are presented in the bottom half of each figure, with drug response (standardised to the median of the control group) on the y-axis and drug concentration on the x-axis. Each dot corresponds to a replicate (typically 3 per dose).

Supplementary Figure S3a -- Patient 294 -- HGSOC -- Stage III

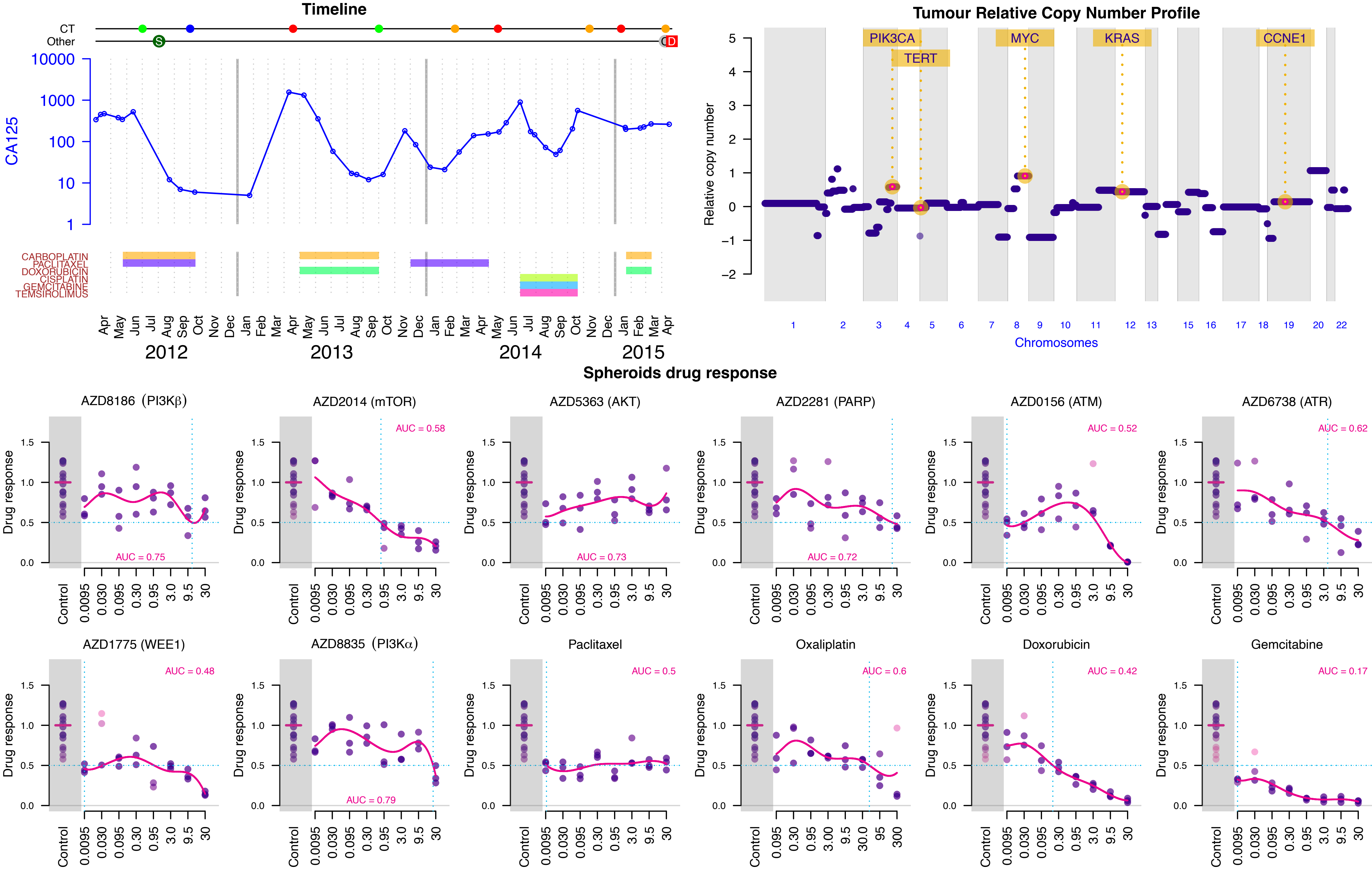

Supplementary Figure S3b -- Patient 333 -- HGSOC -- Stage IV

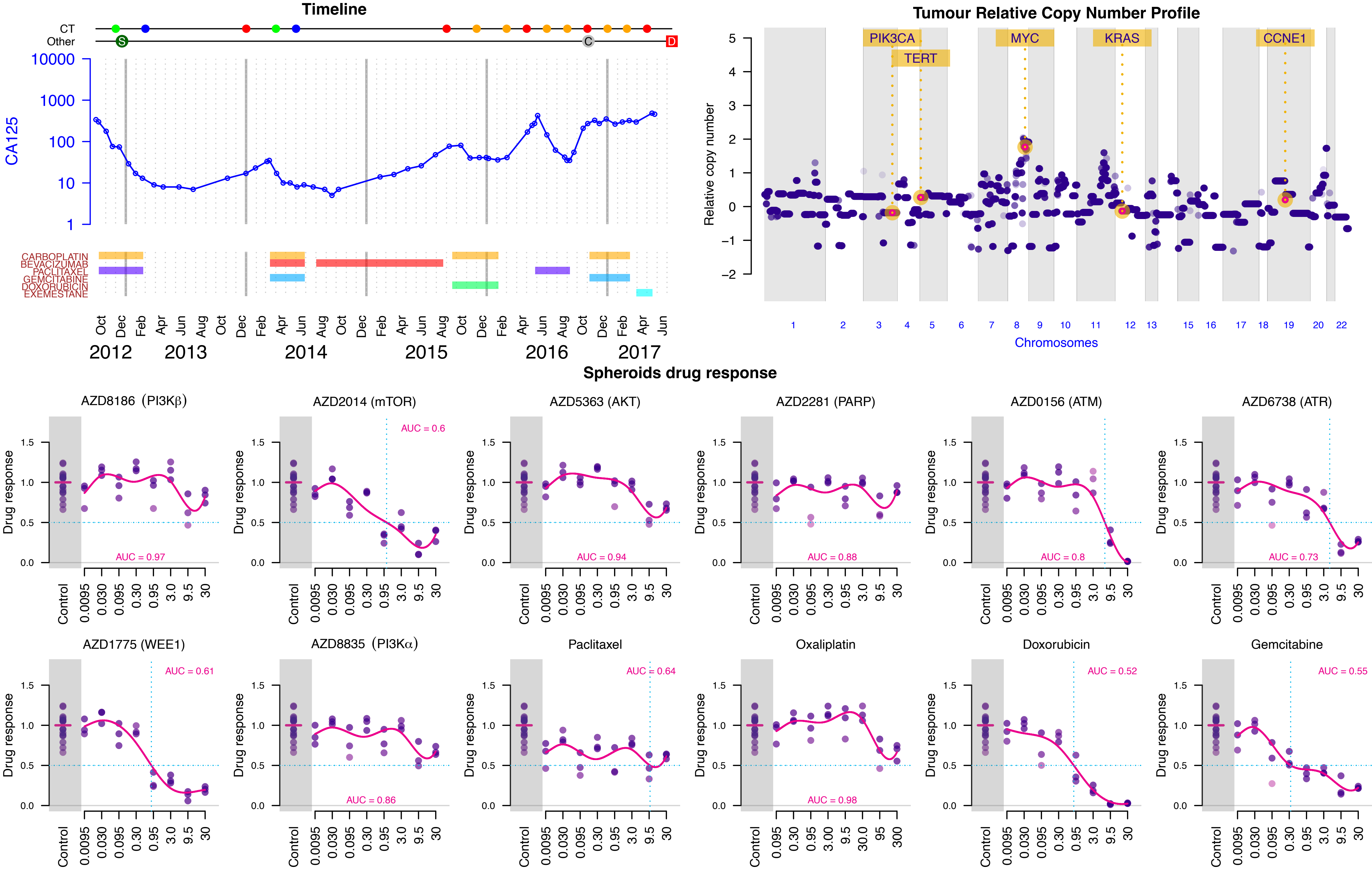

Supplementary Figure S3c -- Patient 364 -- HGSOC -- Stage IV

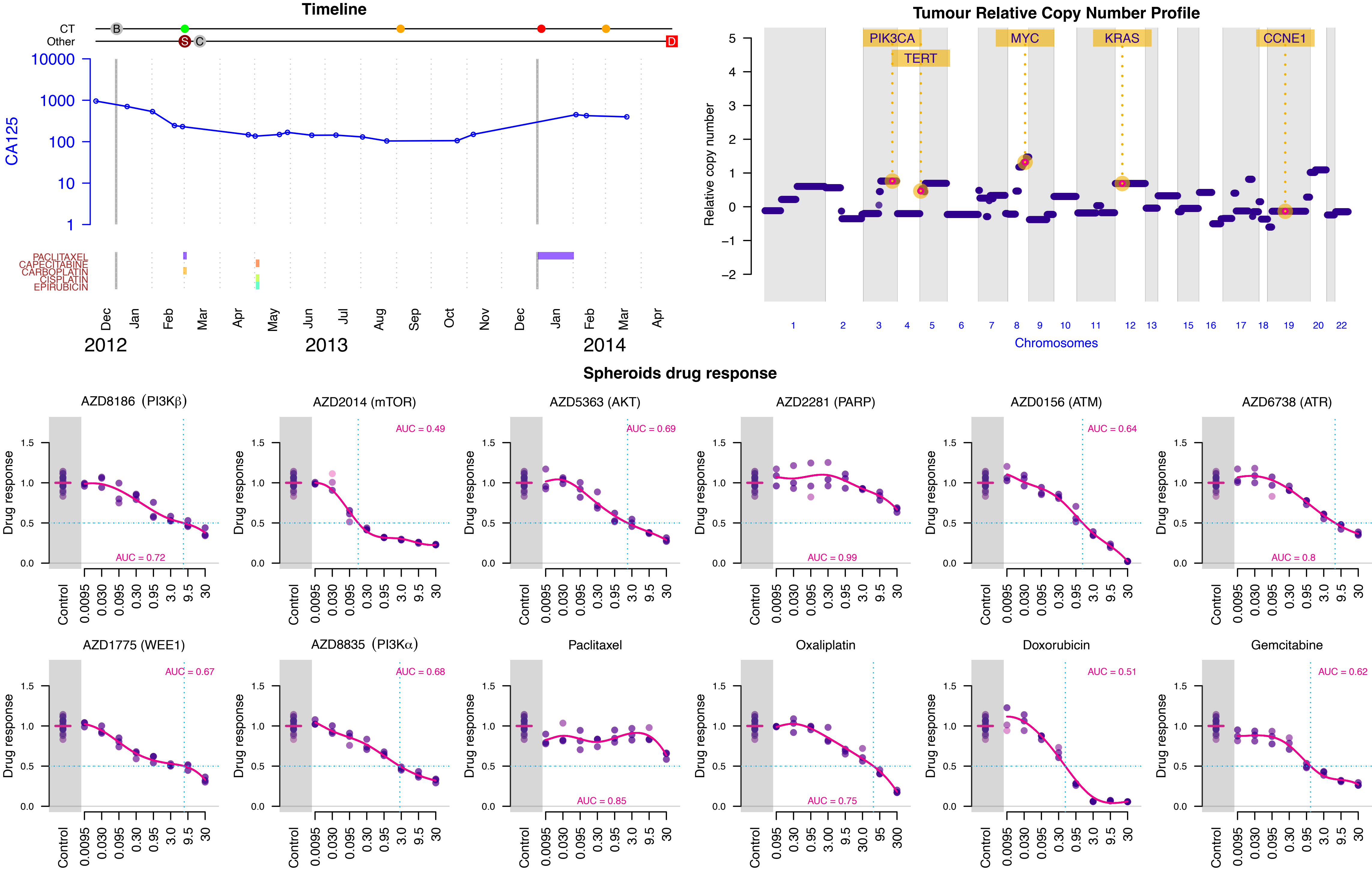

Supplementary Figure S3d -- Patient 409 (First sample) -- HGSOC -- Stage IV

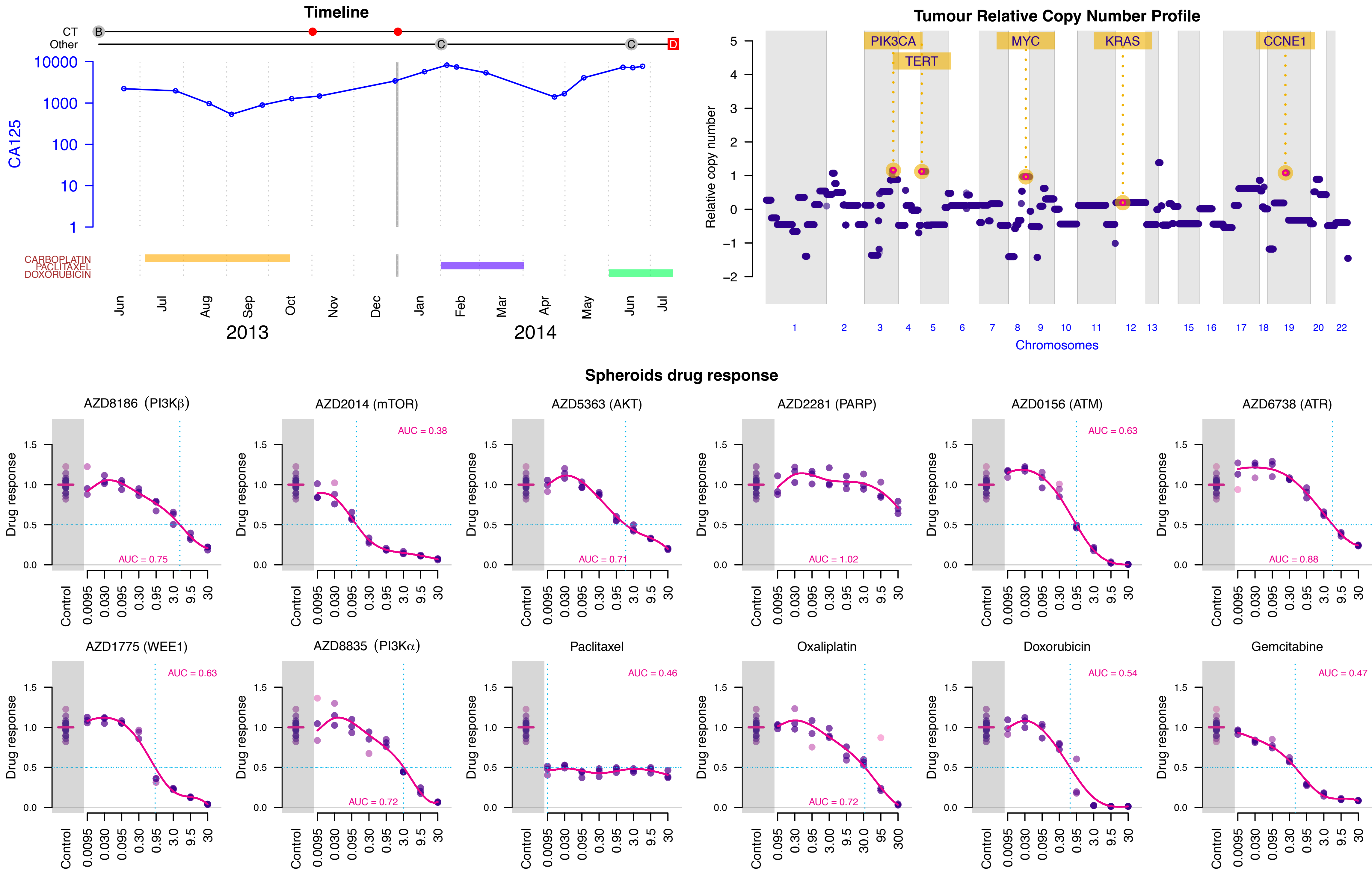

Supplementary Figure S3e -- Patient 409 (Second sample) -- HGSOC -- Stage IV

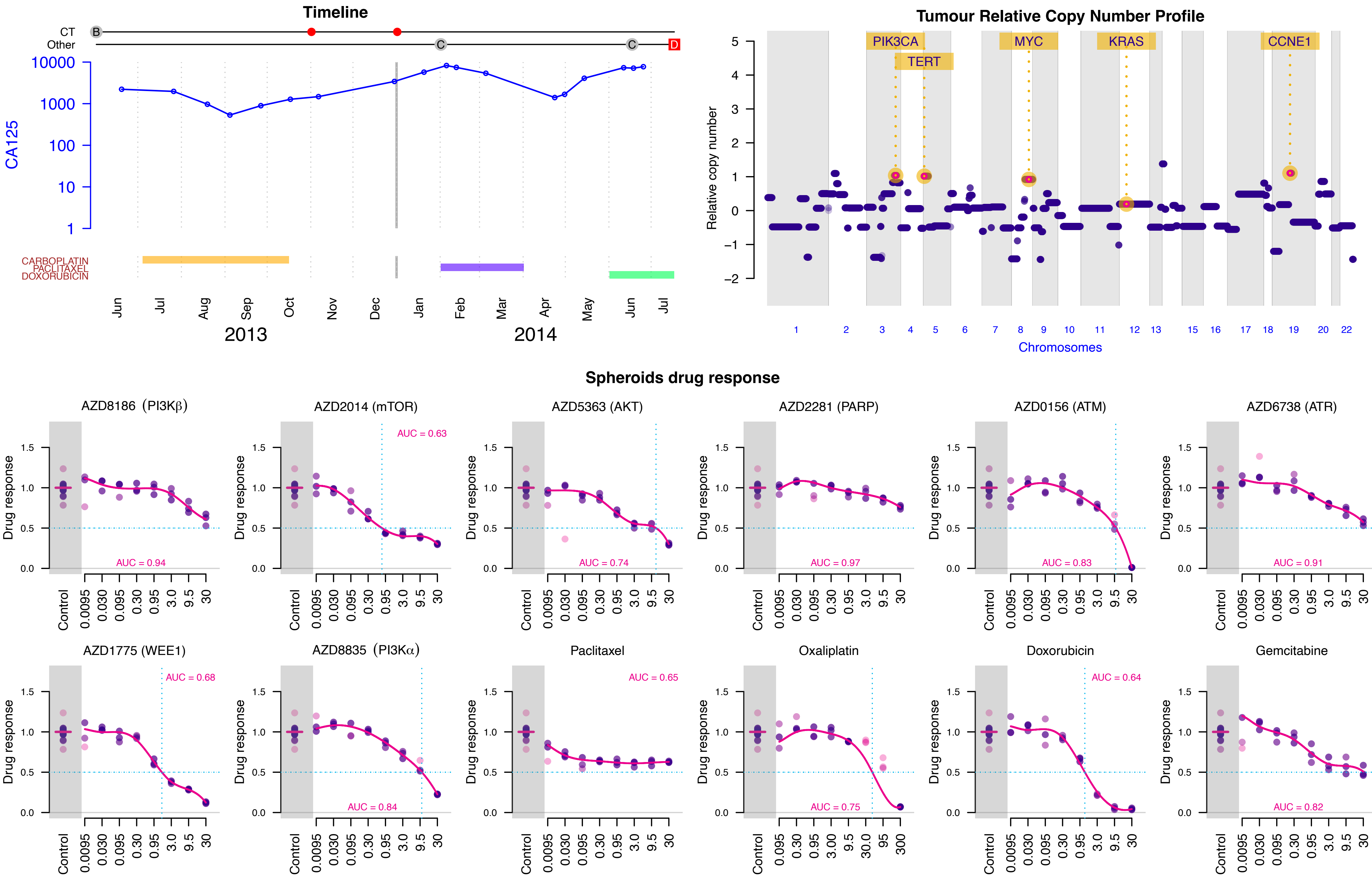

Supplementary Figure S3f -- Patient 413 -- HGSOC -- Stage III

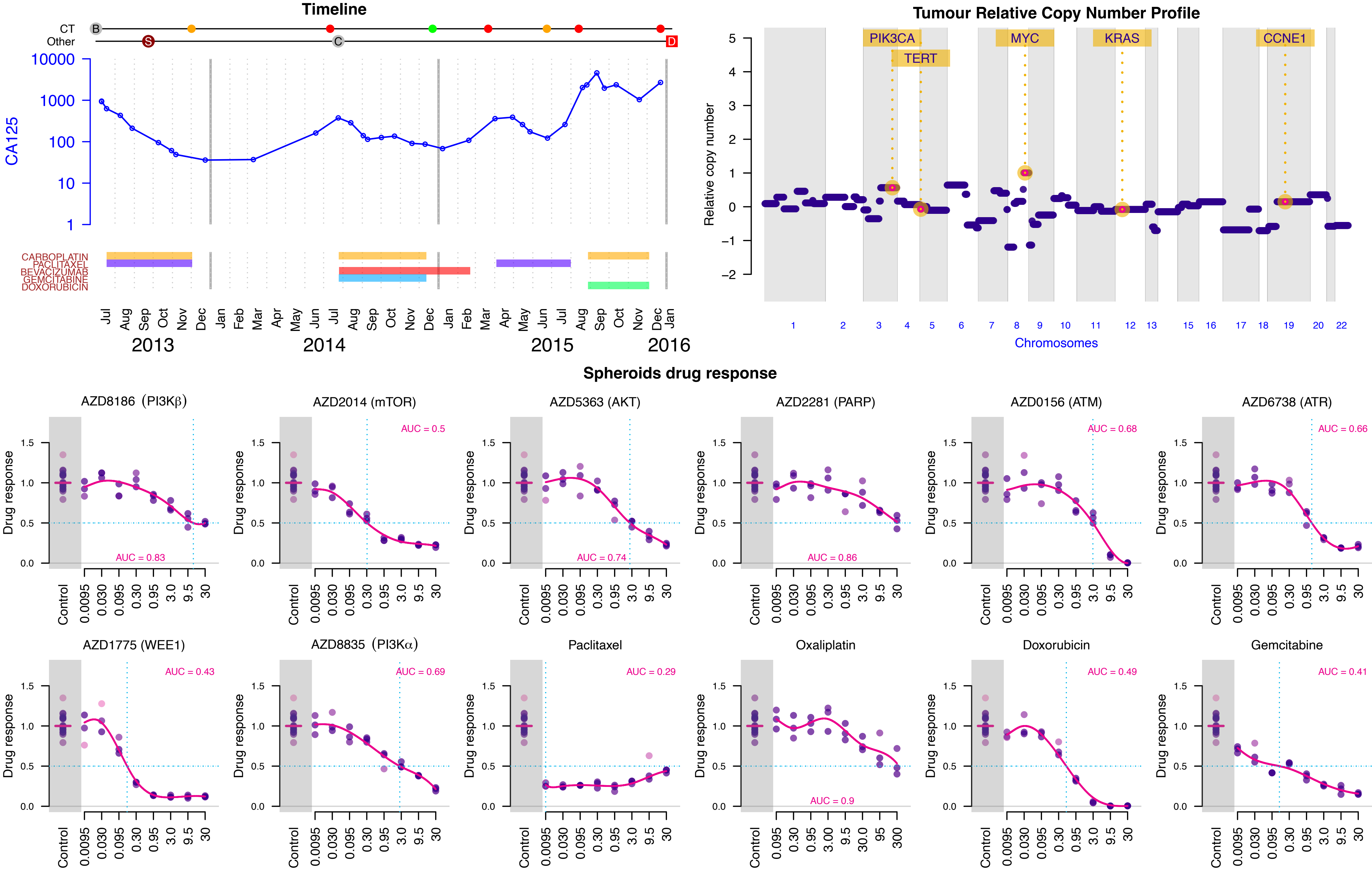

Supplementary Figure S3g -- Patient 466 -- HGSOC -- Stage III

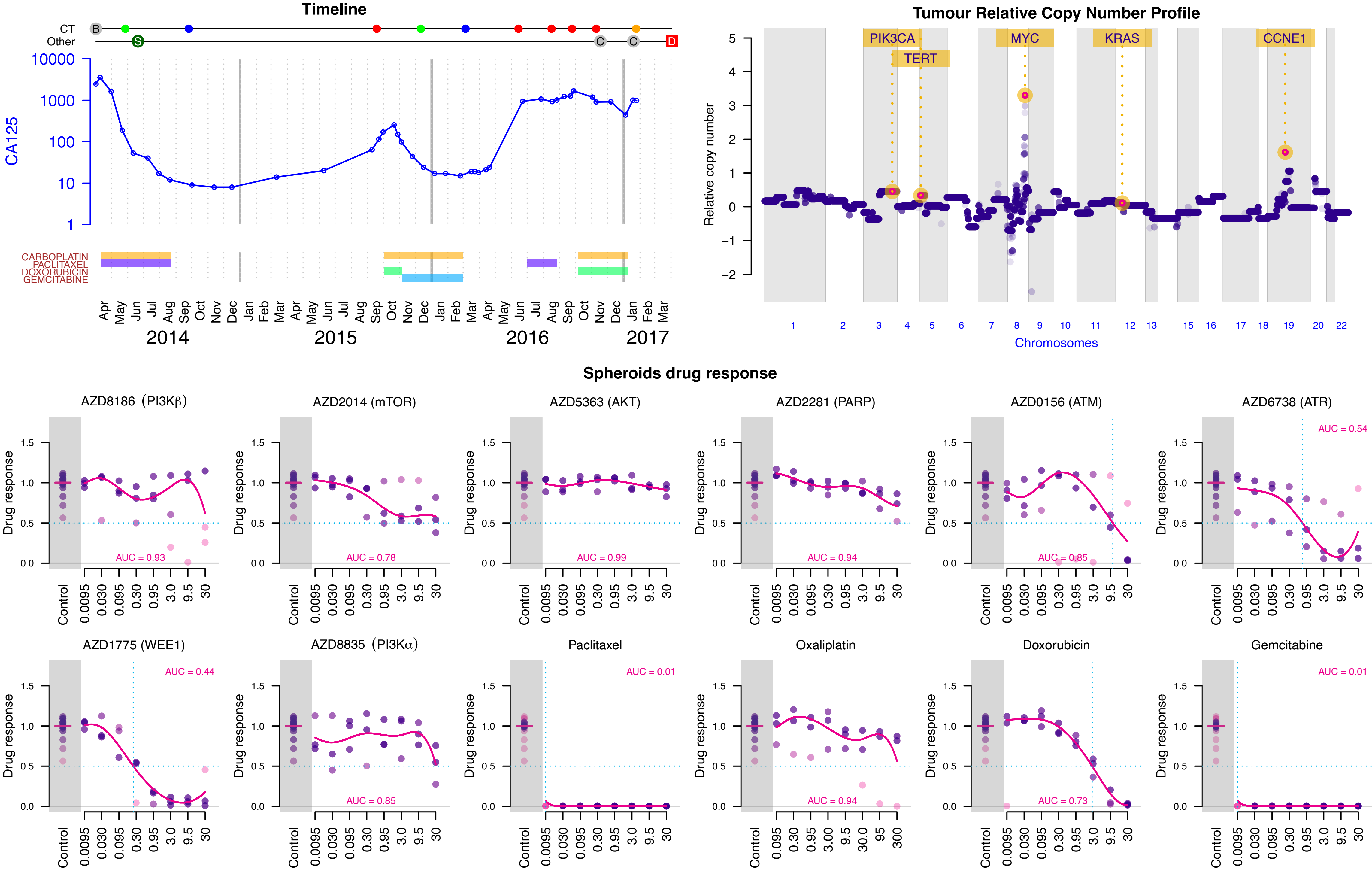

Supplementary Figure S3h –– Patient 467 –– HGSOC –– Stage III

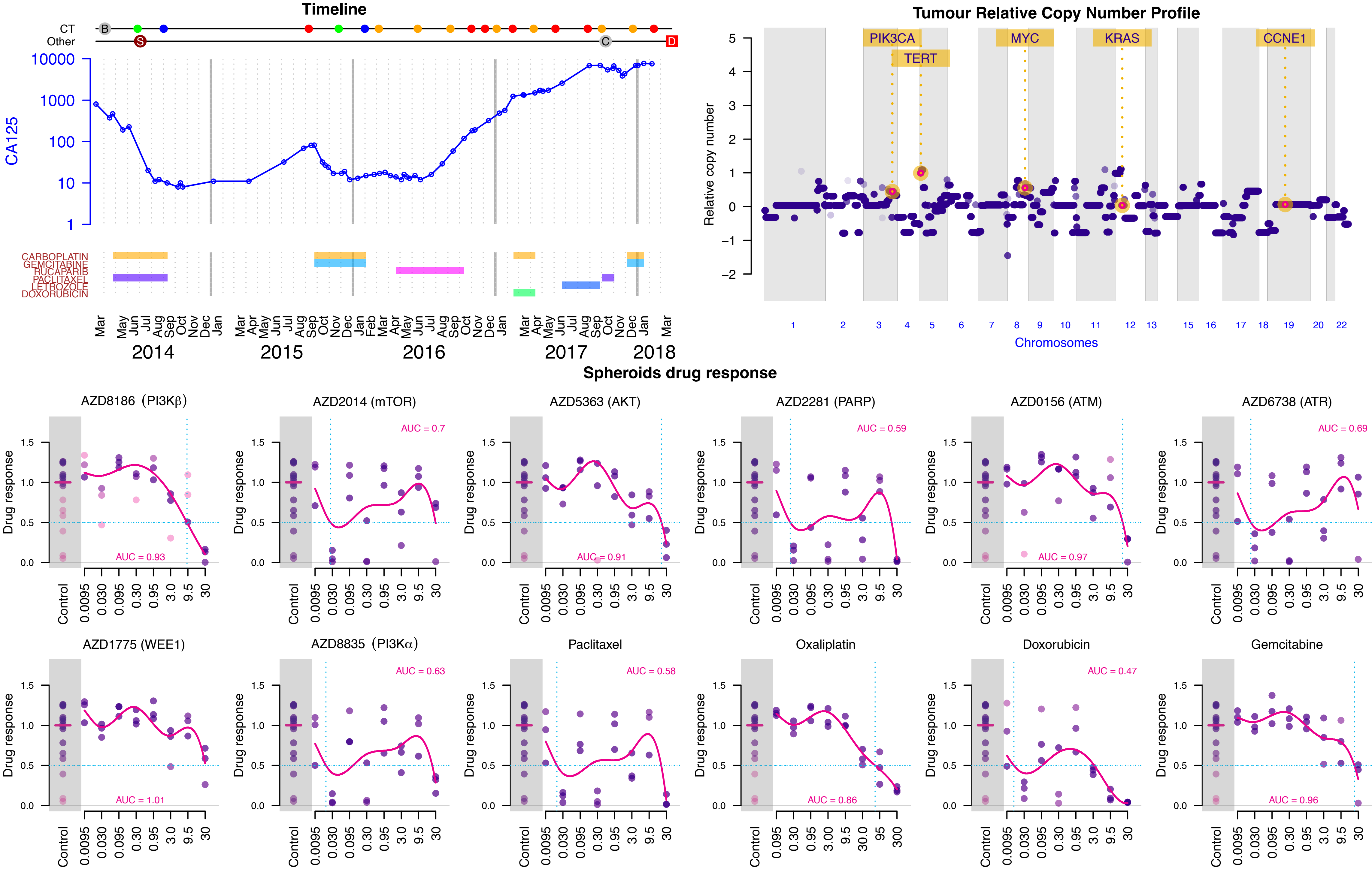

Supplementary Figure S3i -- Patient 525 -- HGSOC -- Stage IV

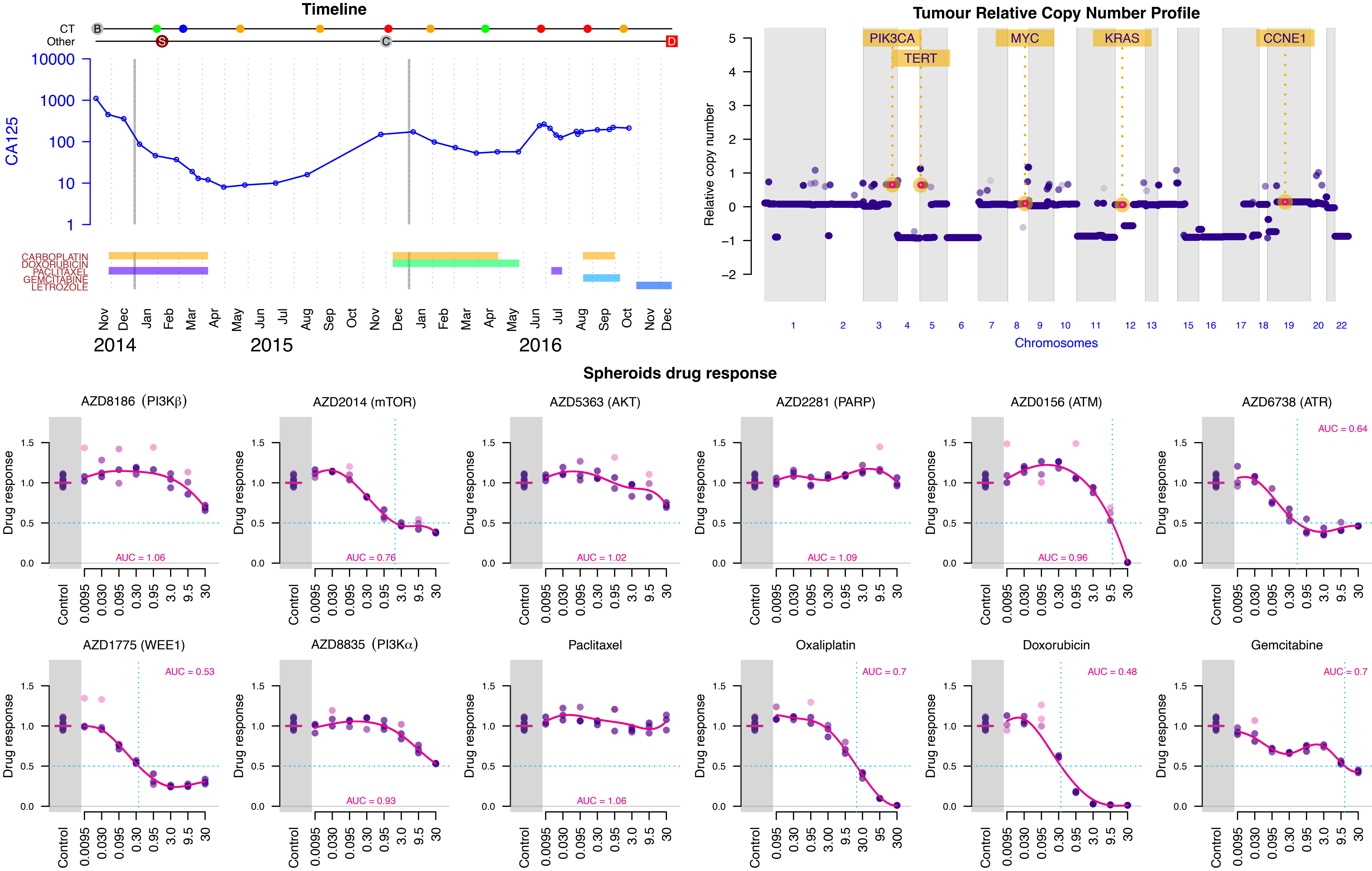

Supplementary Figure S3j -- Patient 626 -- HGSOC -- Stage III

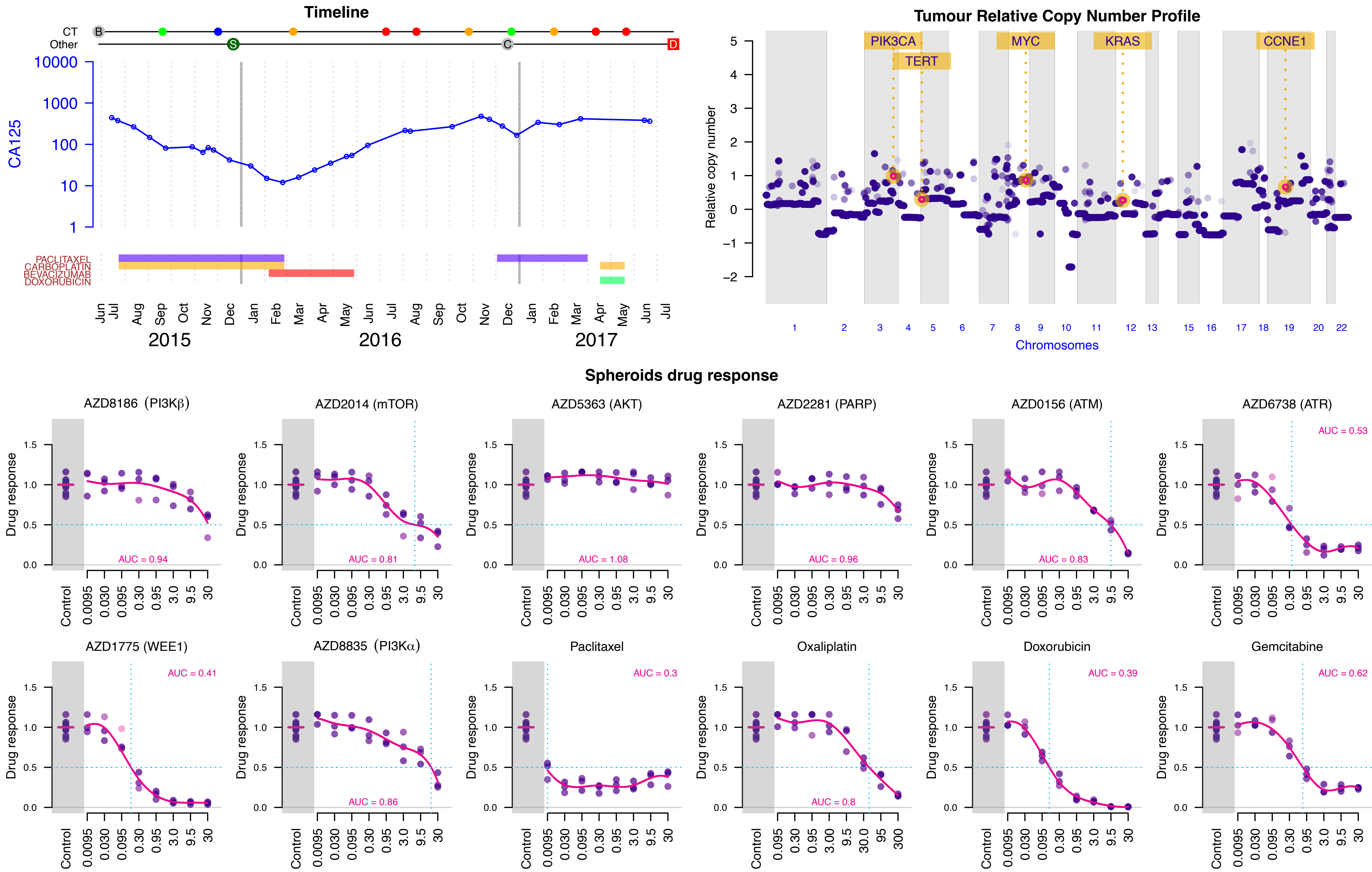

Supplementary Figure S3k –– Patient 648 –– HGSOC –– Stage III

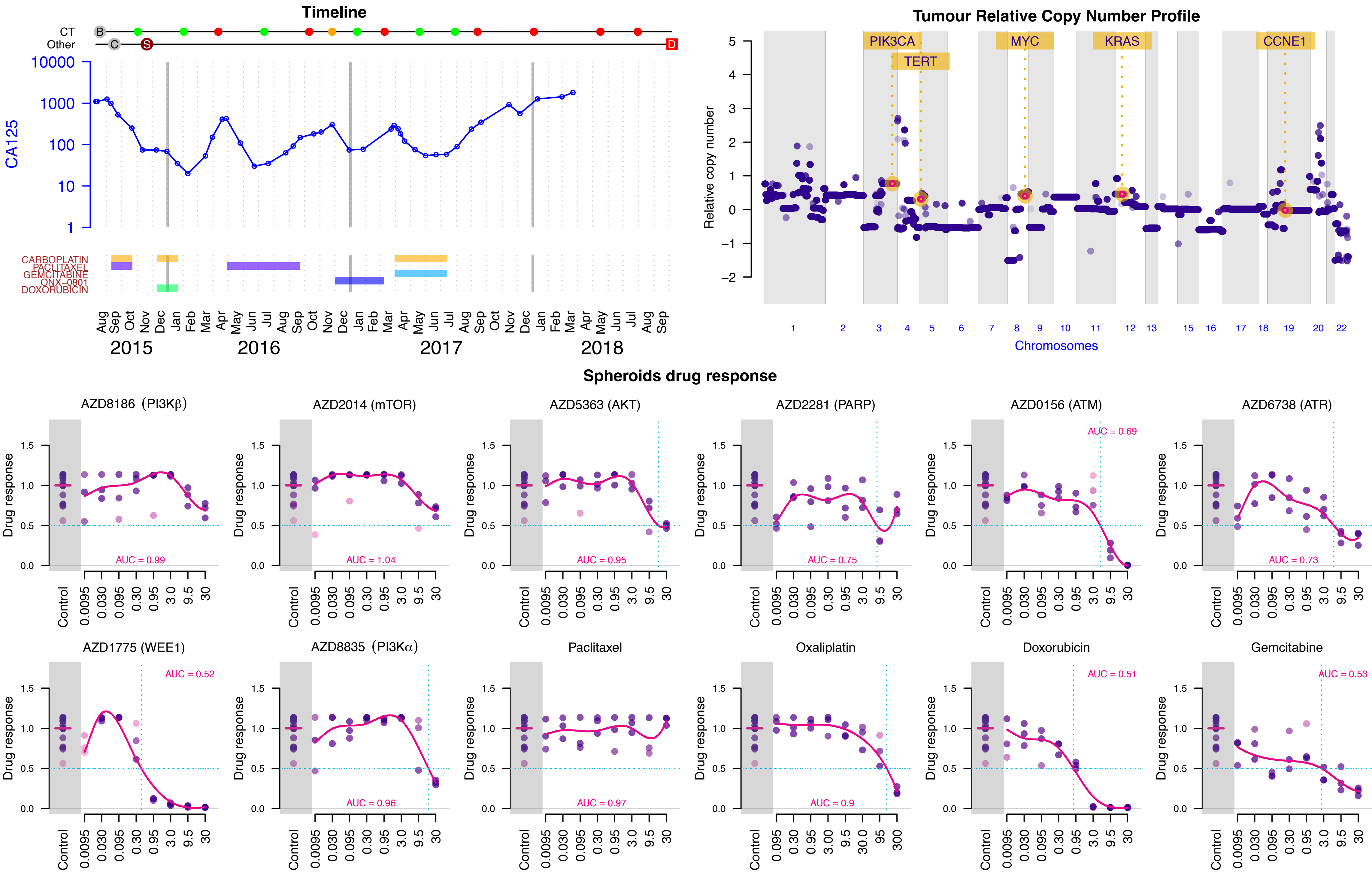

Supplementary Figure S3I -- Patient 669 -- HGSOC -- Stage IV

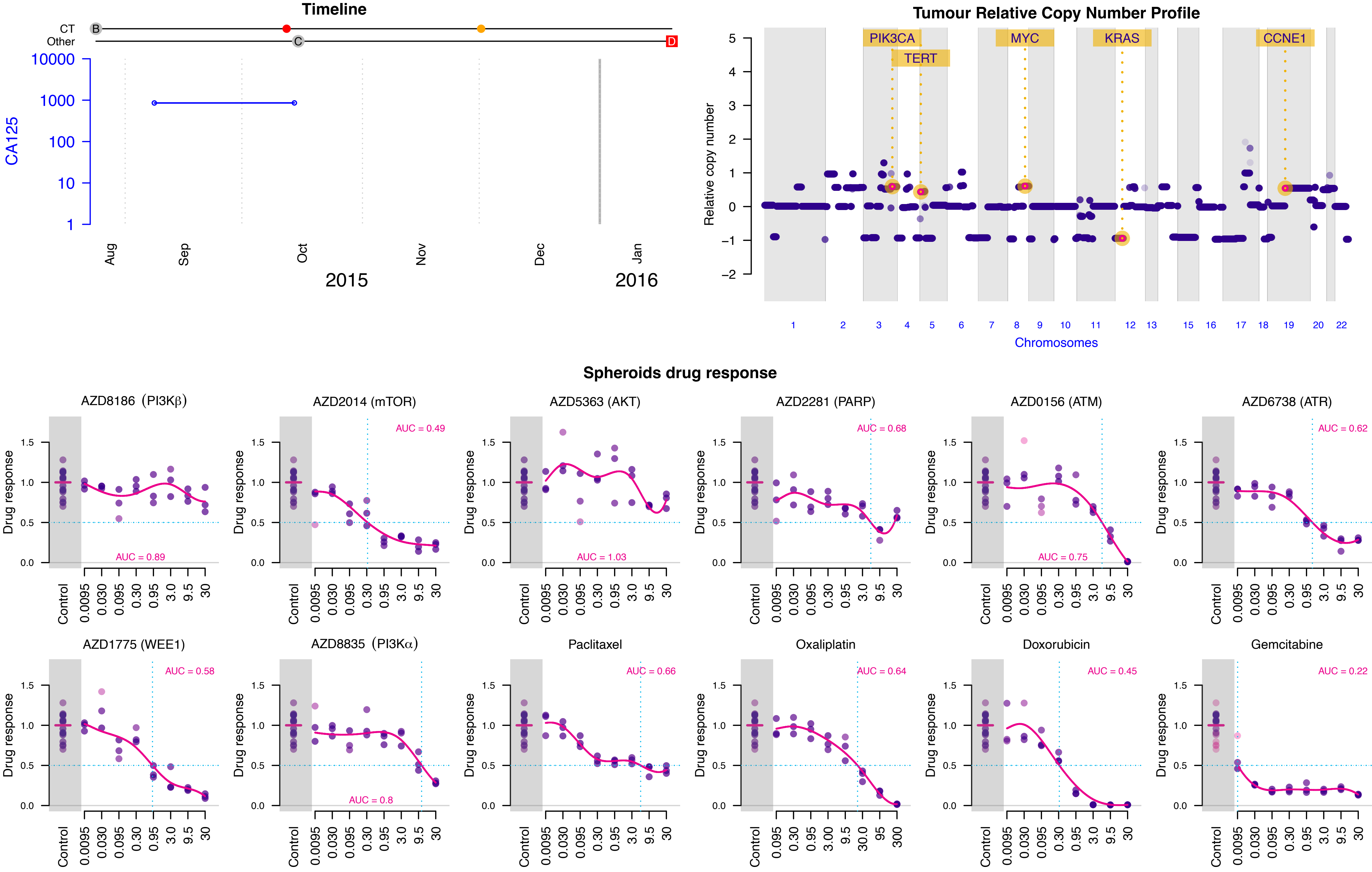

Supplementary Figure S3m -- Patient 687 -- HGSOC -- Stage III

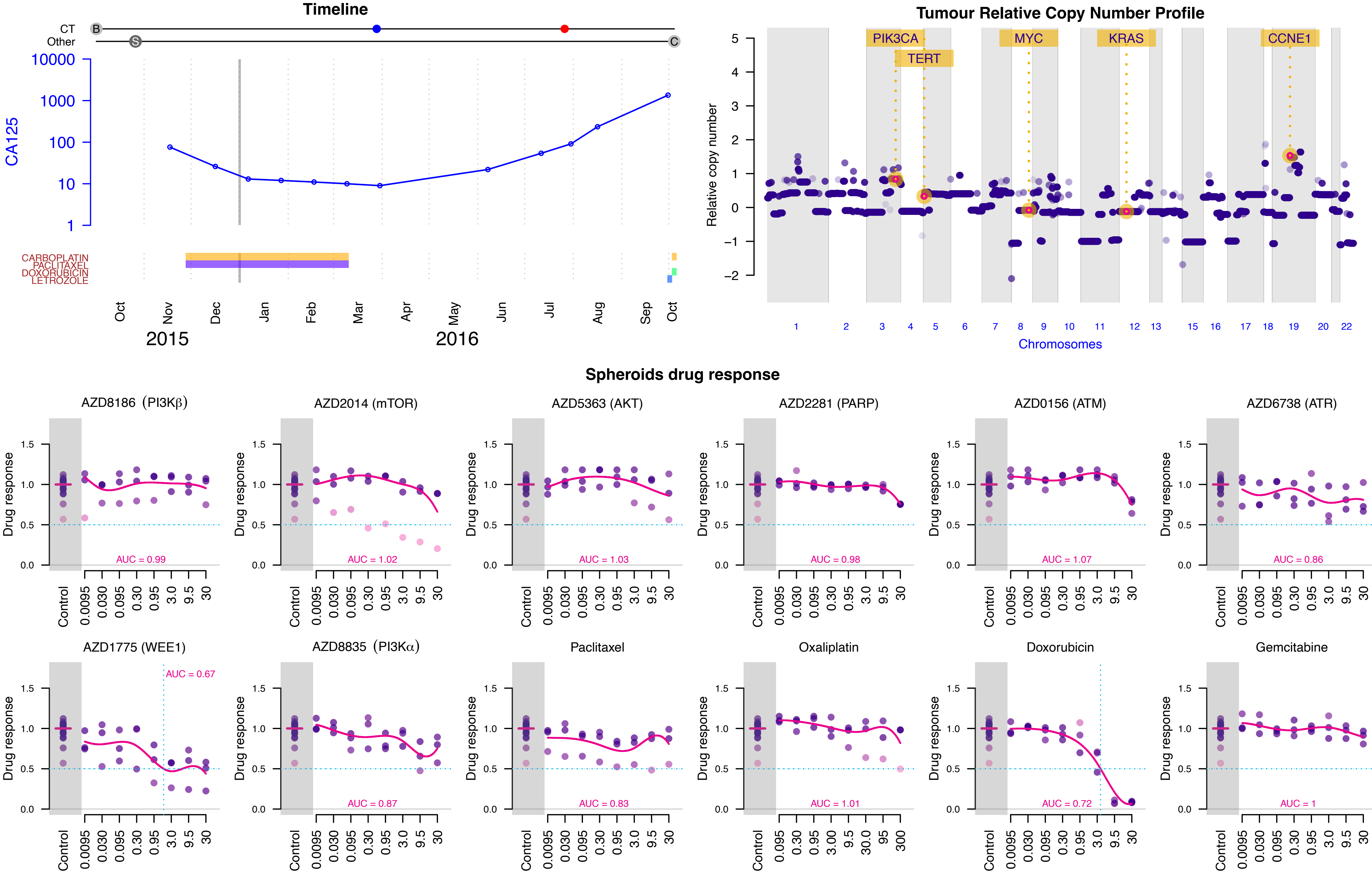

Supplementary Figure S3n -- Patient 720 -- HGSOC -- Stage III

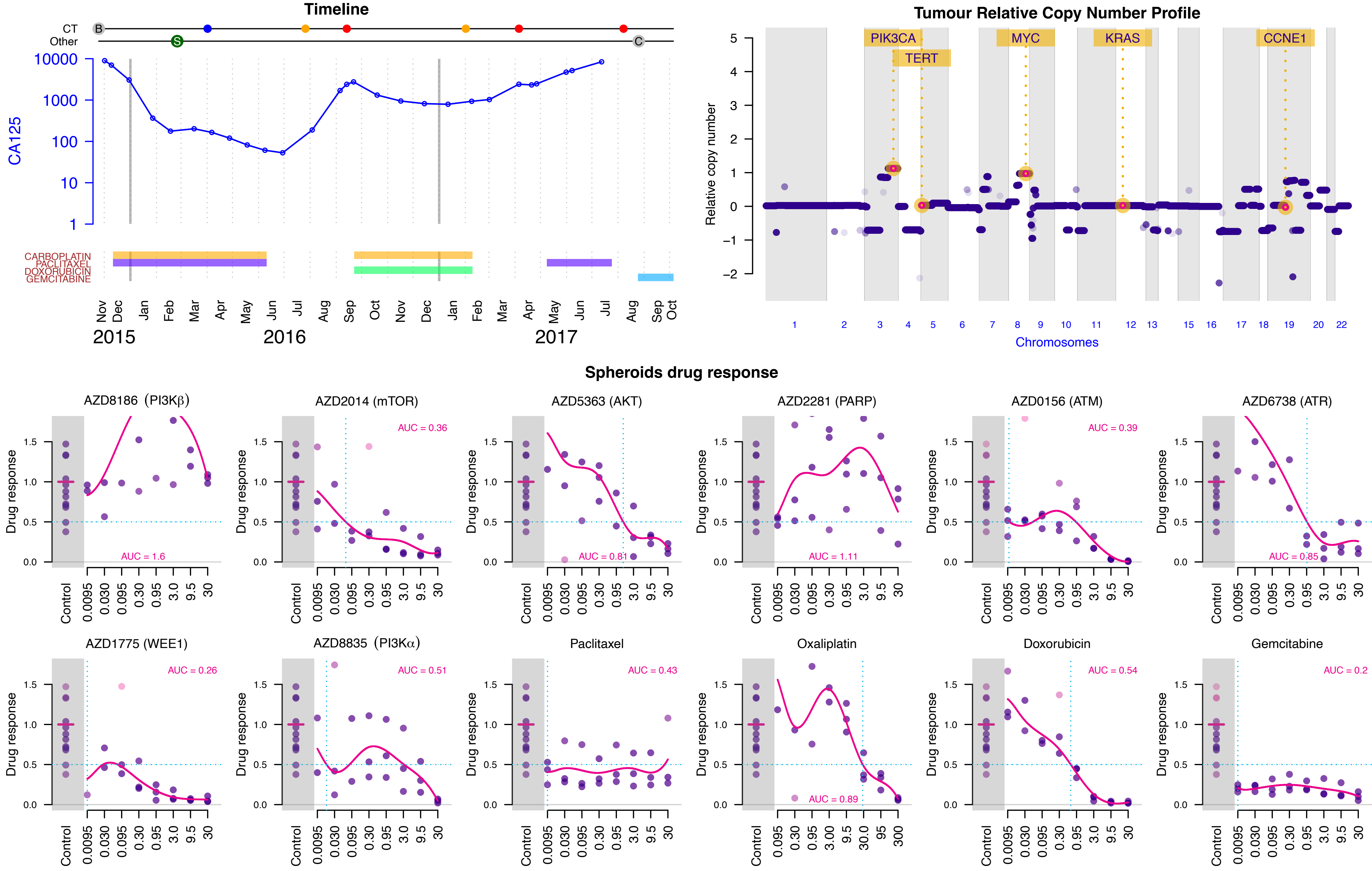

Supplementary Figure S3o -- Patient 783 -- HGS undifferentiated -- Stage NA

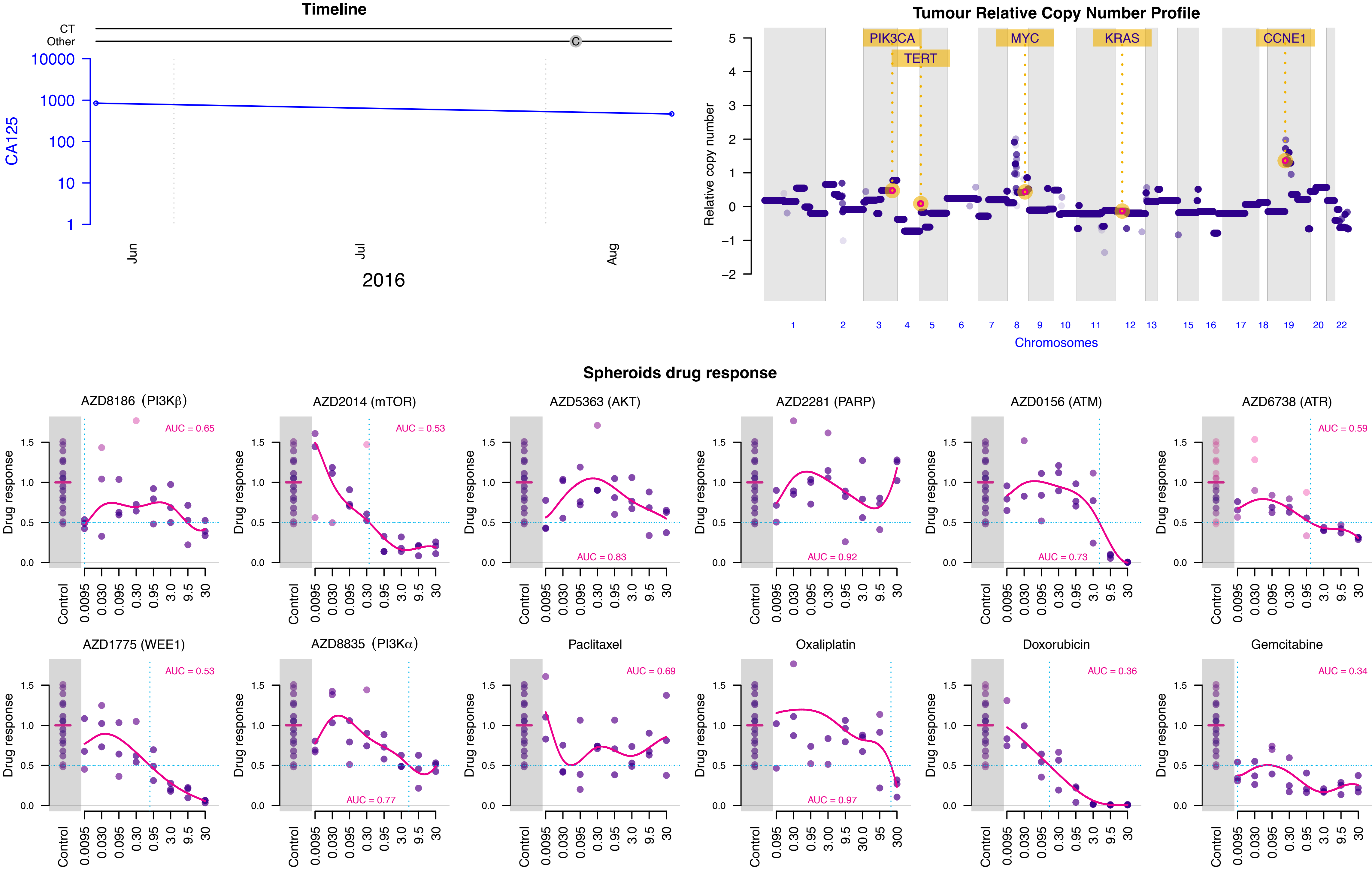

Supplementary Figure S3p -- Patient 788 -- HGSOC -- Stage IV

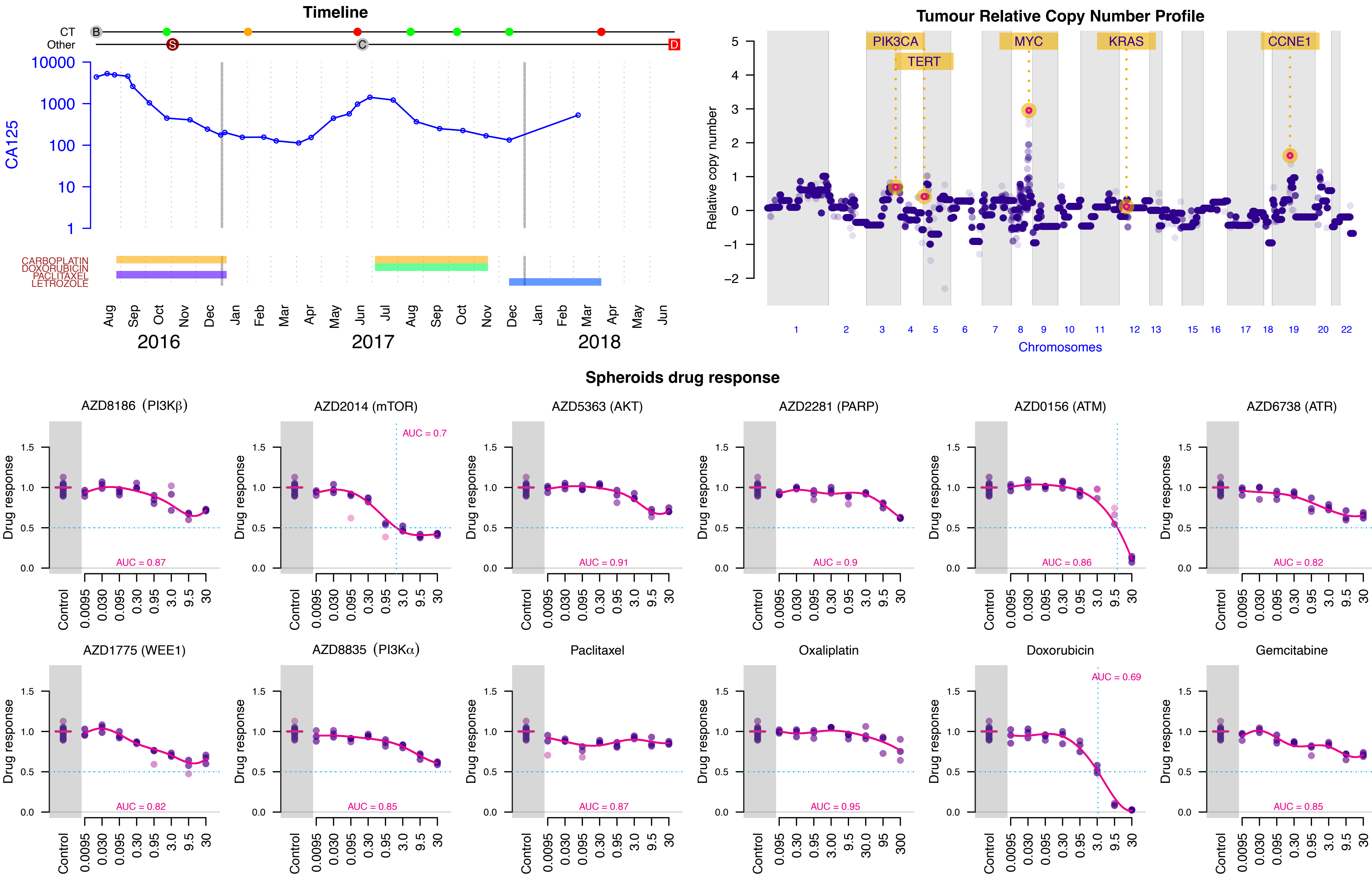

Supplementary Figure S3q –– Patient 800 –– HGS Peritoneum –– Stage III

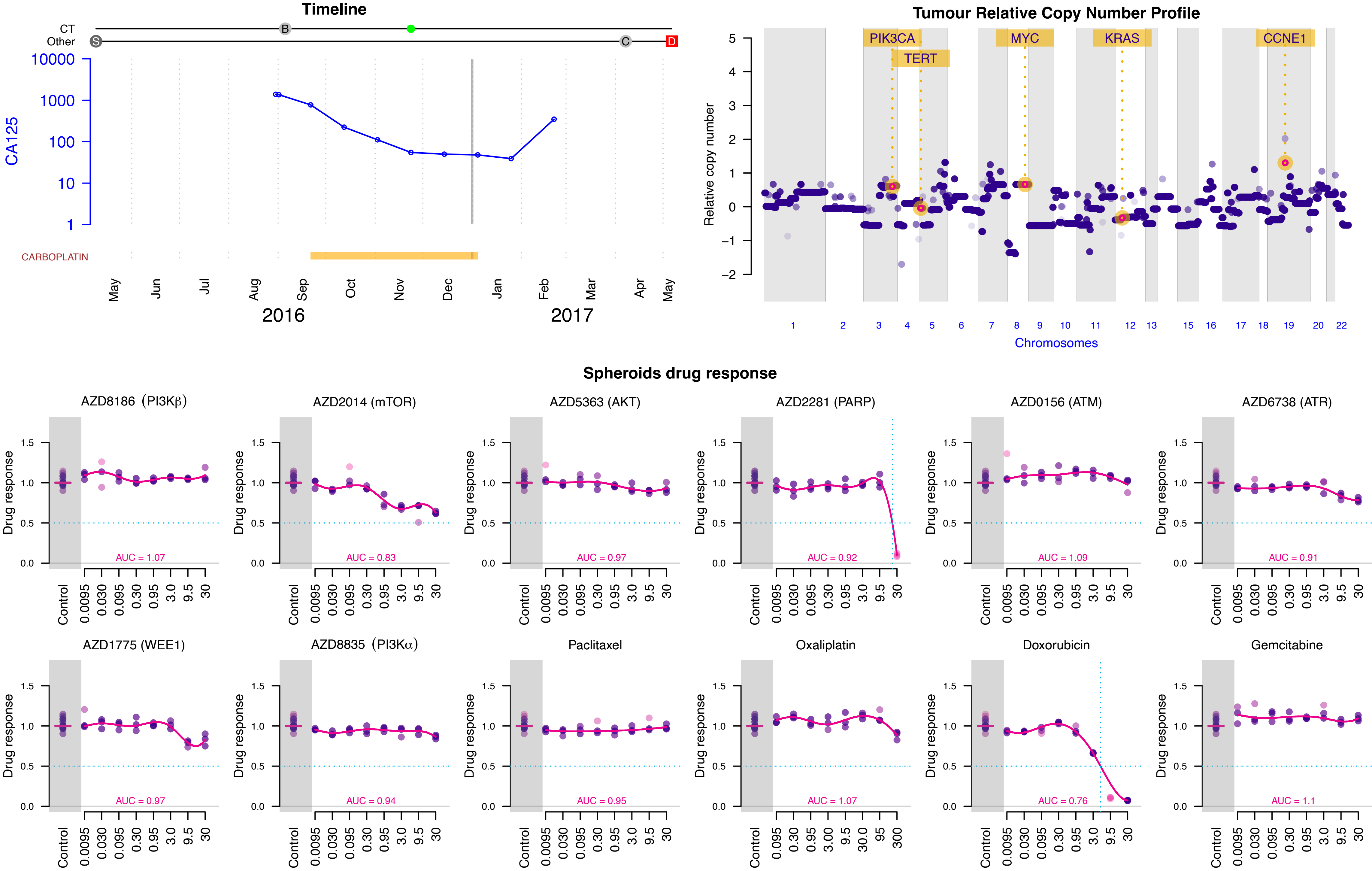

Supplementary Figure S3r -- Patient 819 -- HGSOC -- Stage III

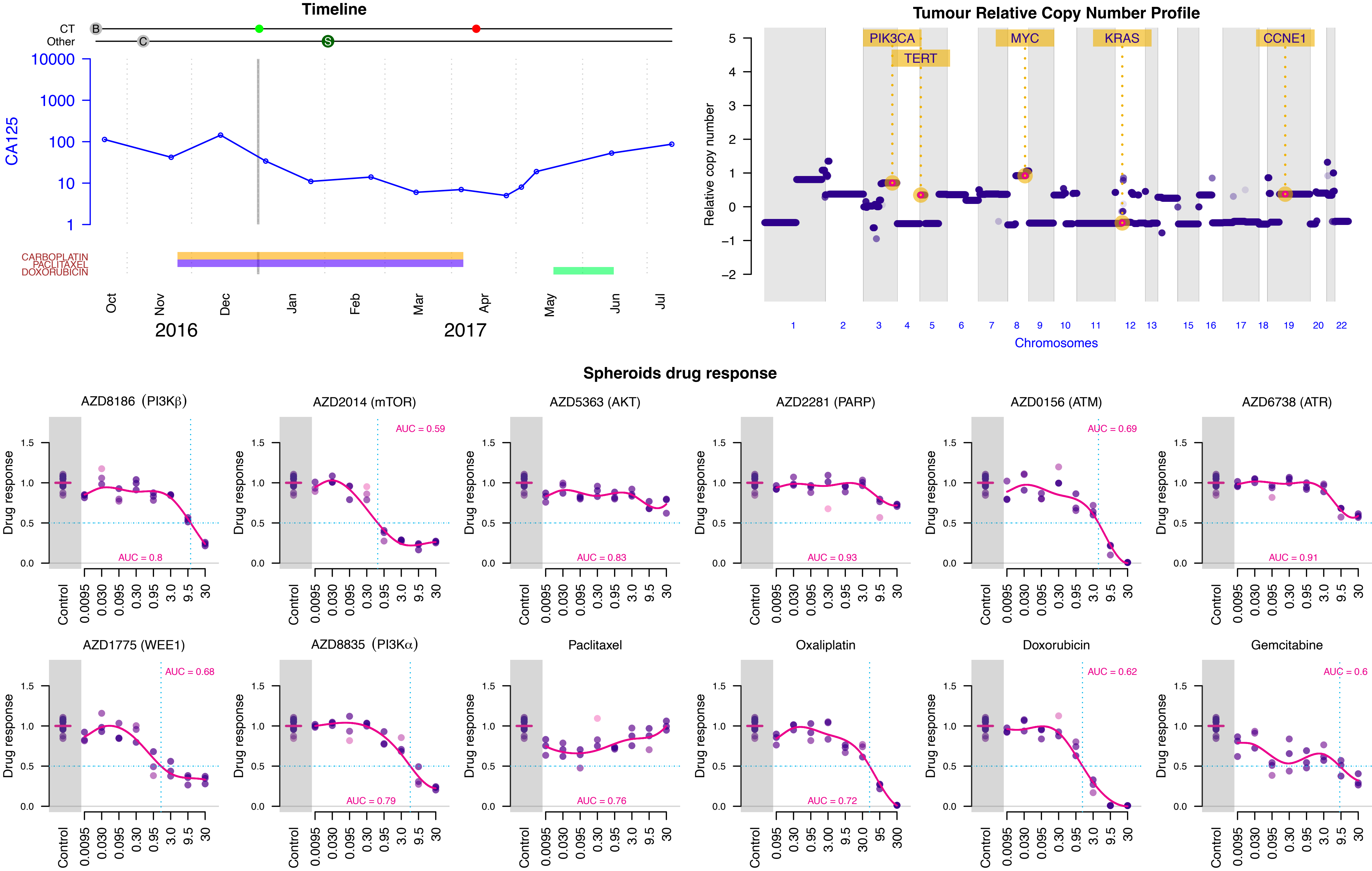

Supplementary Figure S3s -- Patient 839 -- HGSOC -- Stage III

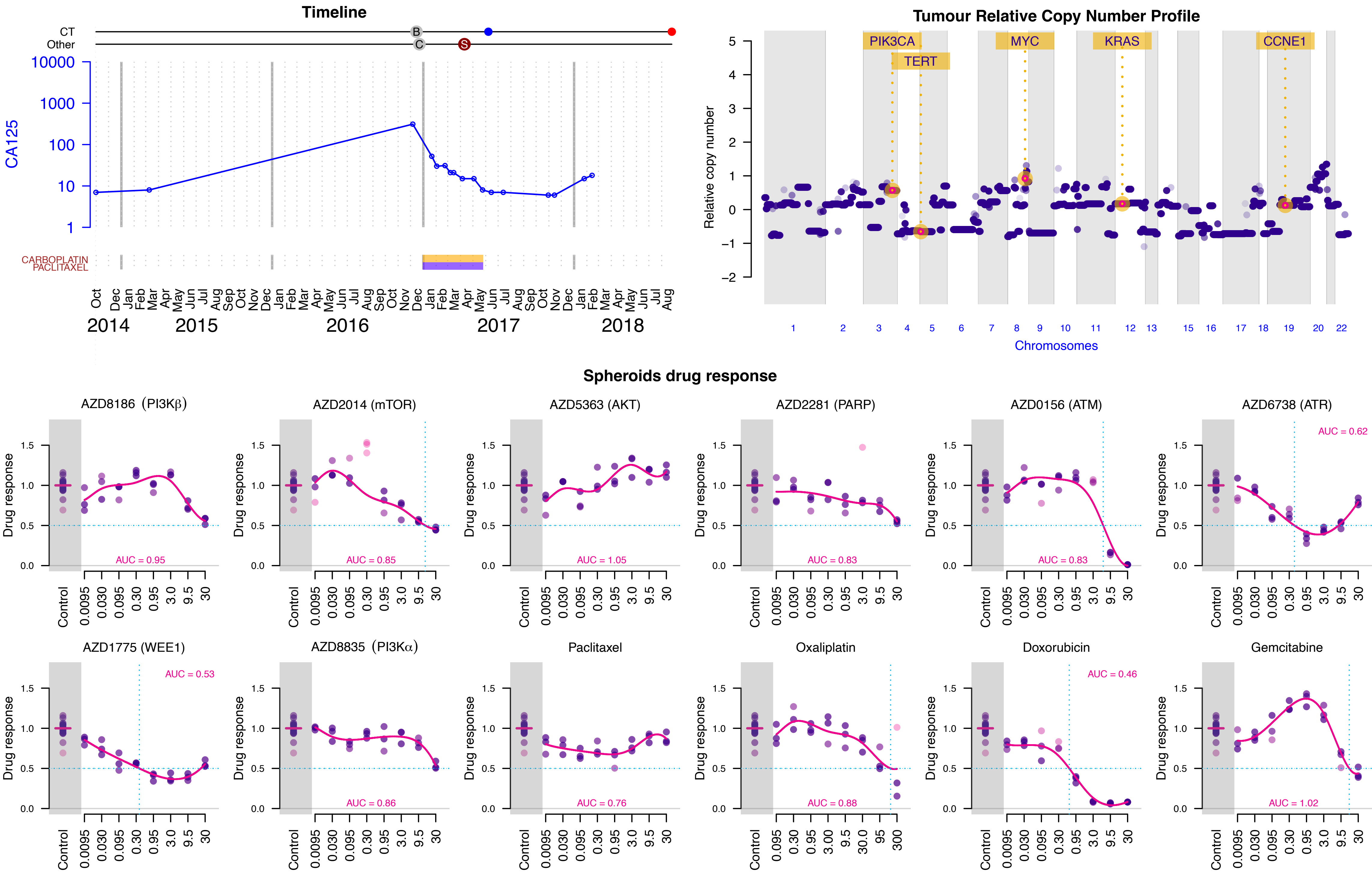

Supplementary Figure S3t –– Patient 864 –– HGS Peritoneum –– Stage III

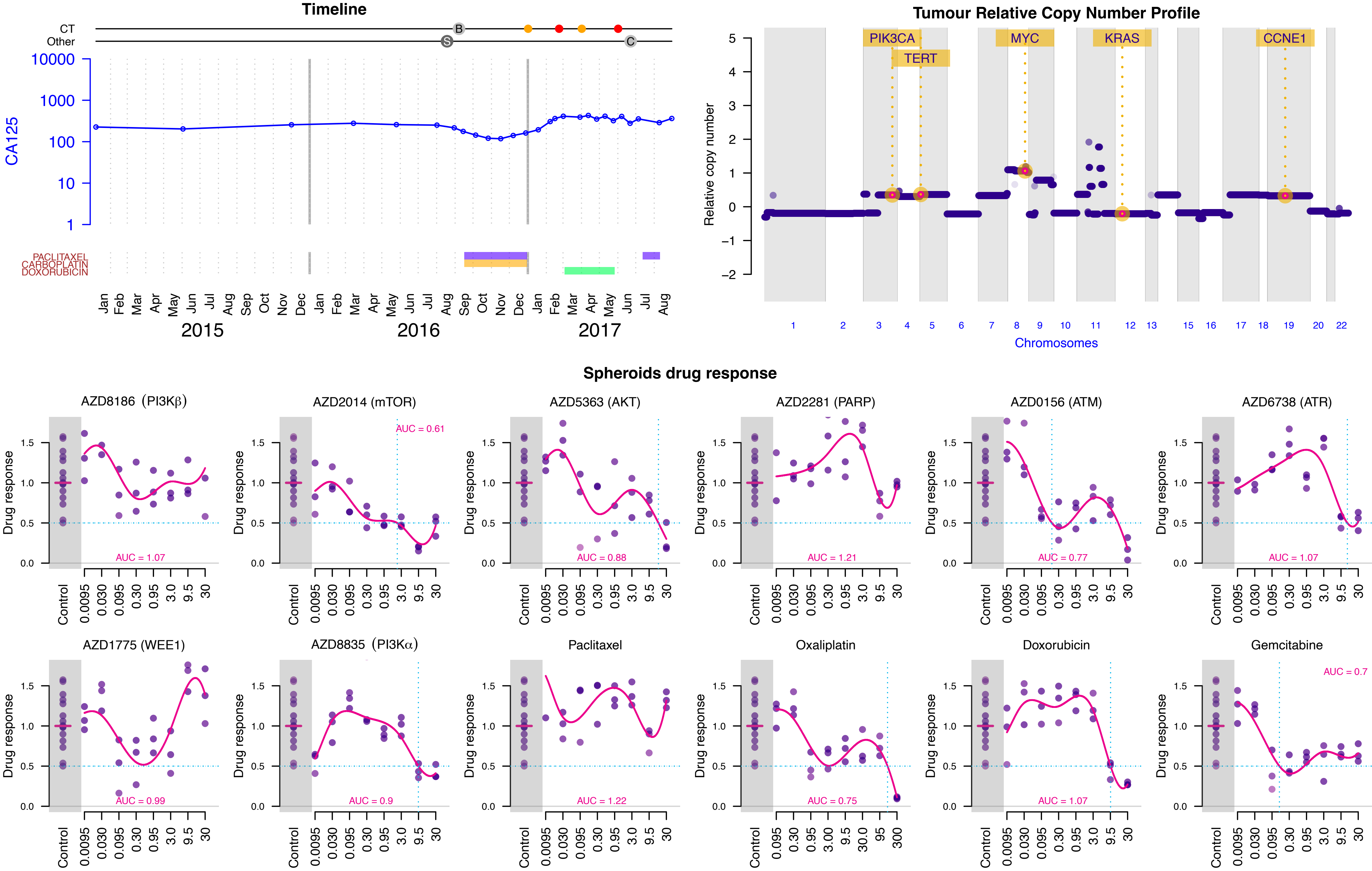

Supplementary Figure S3u –– Patient 875 –– HGSOC –– Stage III

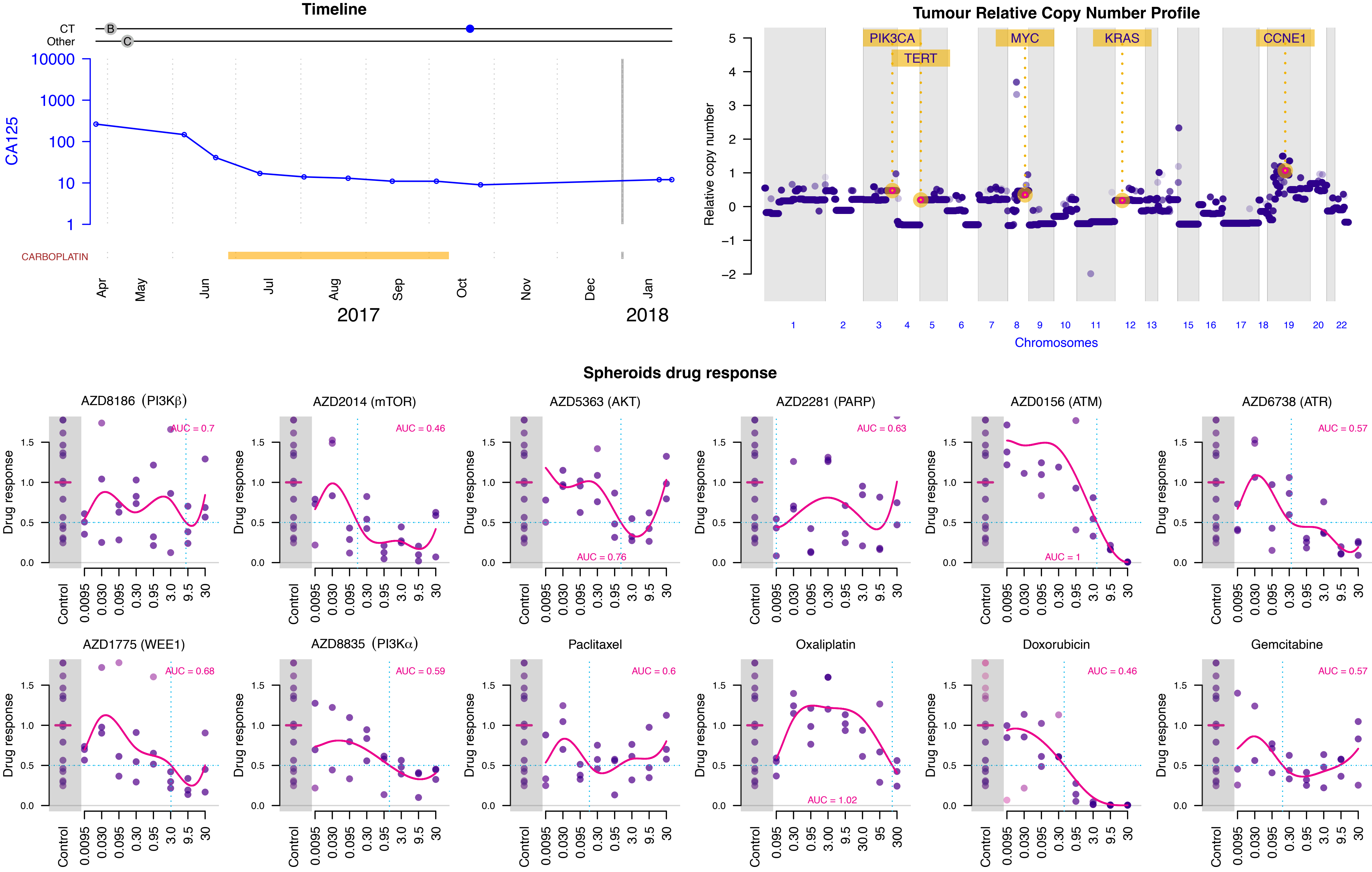

Supplementary Figure S4

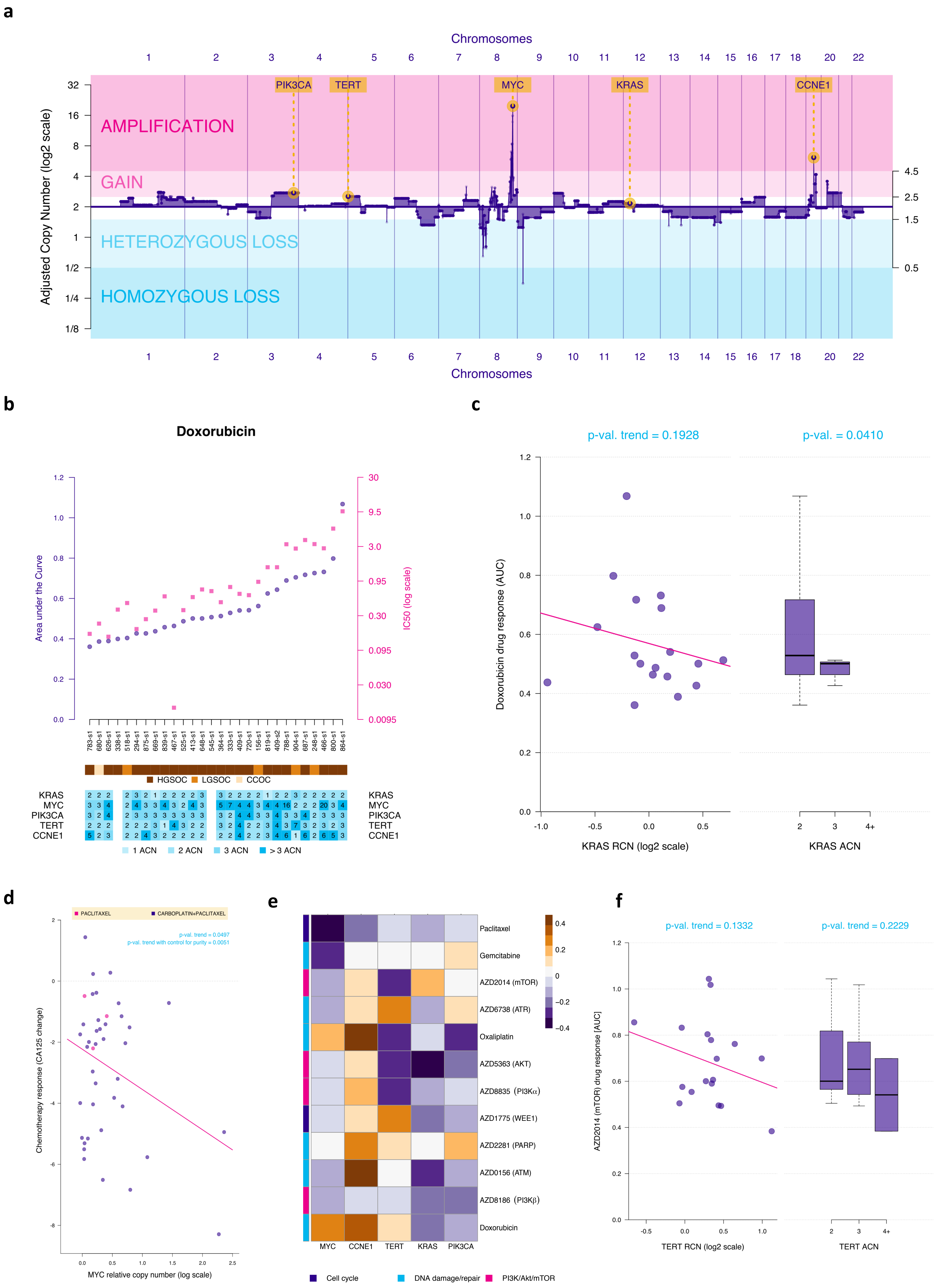

Chromosomal copy number in specific driver genes as predictors of therapeutic response. a. Plot showing an example of genomic profile (adjusted copy number for each gene) for one HGSOC sample (patient 466). **b.** Scatterplot showing doxorubicin response measured in all samples (n=28) following the same format as in panel 2a. **c.** Association between KRAS RCN and ACN and doxorubicin *in vitro* response (as in panel 2b); number of independent samples (n=18) and statistical test identical to Figure 2b; regression effect size of -0.2. **d.** Scatterplot showing the clinical response to paclitaxel (alone and in combination with carboplatin) as a function of MYC RCN. The linear mixed model p-value for the trend (two-sided Wald t-test of the slope parameter) obtained with or without correcting for purity are indicated. Clinical details on the samples used are summarised in Supplementary Table 1 on the right column ("Tumours"). **e.** Heatmap showing the correlation between copy number in selected genes and drug response measured by AUC. The tricoloured bar on the left shows the pathways affected by each specific drug. **f.** Association between TERT RCN and ACN and *in vitro* response to mTOR inhibition (as in panel 2b); number of independent samples (n=18) and statistical test identical to Figure 2b; regression effect size of -0.28. For all boxplots (c and f), the central box was defined by the quantiles 0.25, 0.5 and 0.75 of the data, and the maximum whisker size equals 1.5 times of the interquartile range.

Supplementary Figure S5

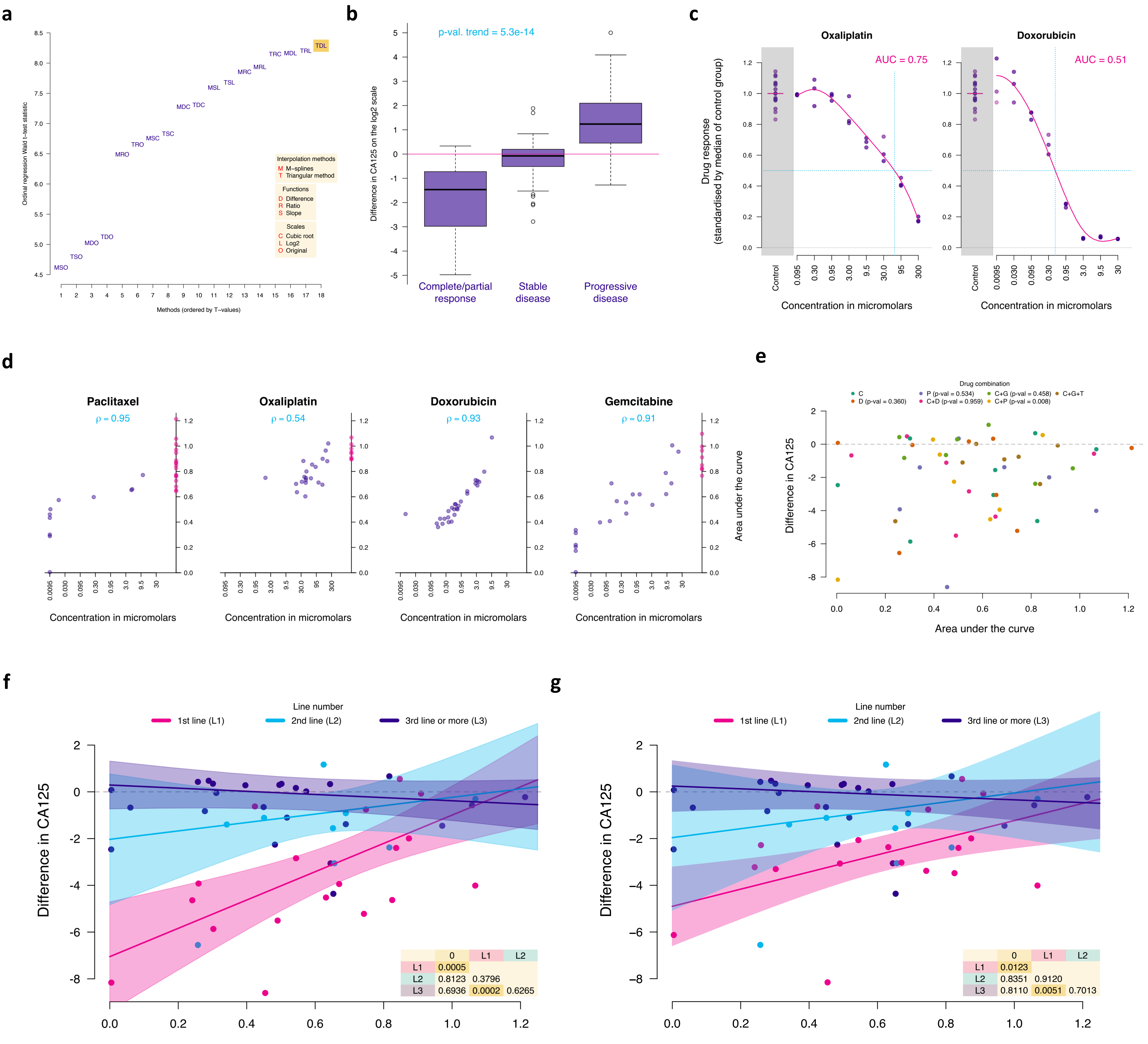

Ex-vivo drug response in primary spheroid samples is associated with clinical response to chemotherapy. Clinical response measures have been previously categorized semi-quantitatively (29). In order to define a continuous variable that integrated all parameters of the degree of response, we compared variation in serum Cancer Antigen 125 (CA125) levels with radiological response on CT scans during treatment, as surrogates of histological response to chemotherapy (Supplementary Figures S3a-u). **a.** Analysis to establish the best method, function and scale to assess CT response by using CA125 variation. The difference between levels of CA125 levels measured in log2 scale at the time of CT imaging had the best performance as a numerical predictor of CT-inferred variation in the disease burden **b.** Boxplots showing the association between variation in CA125 (measured by the difference of CA125 values in log 2 scale after and before each chemotherapy cycle) split by categories of CT radiological response and p-value of the two-sided Jonckheere-Terpstra test of association P ≪0.001. N=159 values of CA125 variation from n=27 independent patients. For all boxplots, the central box was defined by the quantiles 0.25, 0.5 and 0.75 of the data, and the maximum whisker size equals 1.5 times of the interquartile range. **c.** Example of *in vitro* response to oxaliplatin and doxorubicin in a spheroid sample. Each dot measures cell viability against the average cell viability of the controls for each drug concentration after 5 days incubation. The pink line corresponds to the M-spline fit. AUC was obtained by integrating the drug response M-spline fit over the range of interest on the log scale. Intersection of dotted blue lines indicates IC50. Dot plot in grey shading indicates control data. We quantified cell viability in 26 primary human-derived spheroid samples after 5 days of *ex vivo* exposure to eight concentrations of each individual drug. The responses to four standard of care drugs (oxaliplatin, paclitaxel, doxorubicin and gemcitabine) were compared to drug-free control spheroid samples: these were reproducible across technical and biological replicates and were not influenced by the length of the drug viability assay. Oxaliplatin was substituted for cisplatin and carboplatin as these are inactivated by DMSO and produces highly similar cellular effects. **d.** Scatterplots showing the association between area under the curve values and IC50 for selected drugs. Each dot represents one sample. Pink dots represent cases where IC50 was not reached owing to viability ≤50% at maximum dose Spearman's Rho correlation estimates are indicated in turquoise. **e & f.** Scatterplots showing the clinical responses measured by variation in logarithmic scale of CA125 during each chemotherapy regimen/line in each patient (y axis) as a function of the in-vitro response to the same drugs, measured by area under the curve on samples from the same patient (x axis). When combinations of drugs were used in the clinical setting, the combined AUC was obtained by multiplying AUC for individual drugs; Points are colour-coded by drugs (panel e) or chemotherapy line numbers (panel f). In panel e, the p-values of the two-sided Wald t-tests defining if the slope parameter of linear mixed models corresponding to each drug are different from 0 are indicated in the legend. In panel f, the mixed model fitted lines per chemotherapy lines and corresponding 95% confidence intervals (shaded areas) are indicated. The p-values of the Wald t-tests performing pairwise comparison of the chemotherapy line slopes or comparing the chemotherapy line slopes to 0 are indicated P: paclitaxel; C: carboplatin; D: doxorubicin; G: gemcitabine; T: targeted inhibitor).When we compared in-vitro response (AUC) to the observed clinical response (CA125 changes) in the same patient to the same drug or class of drugs, we found that the correlation between in vitro and clinical response was strongest for the combination of platinum-based and paclitaxel and first-line chemotherapy. **g.** Plot replicating the analysis in the plot f after excluding CA125 variation in the month after the surgery to eliminate possible effect of surgery in CA125 changes.

Supplementary Figure S6

a

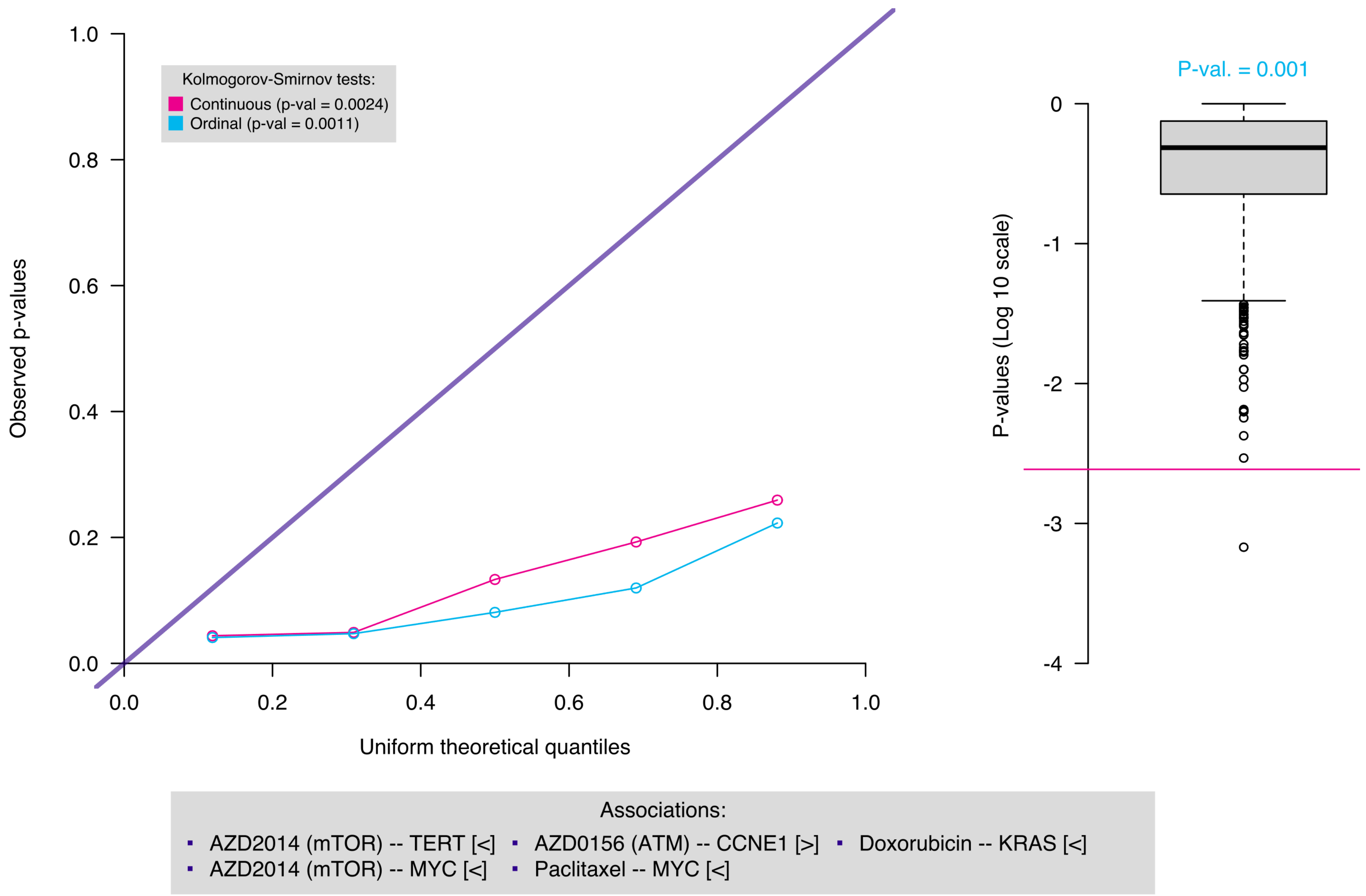

b

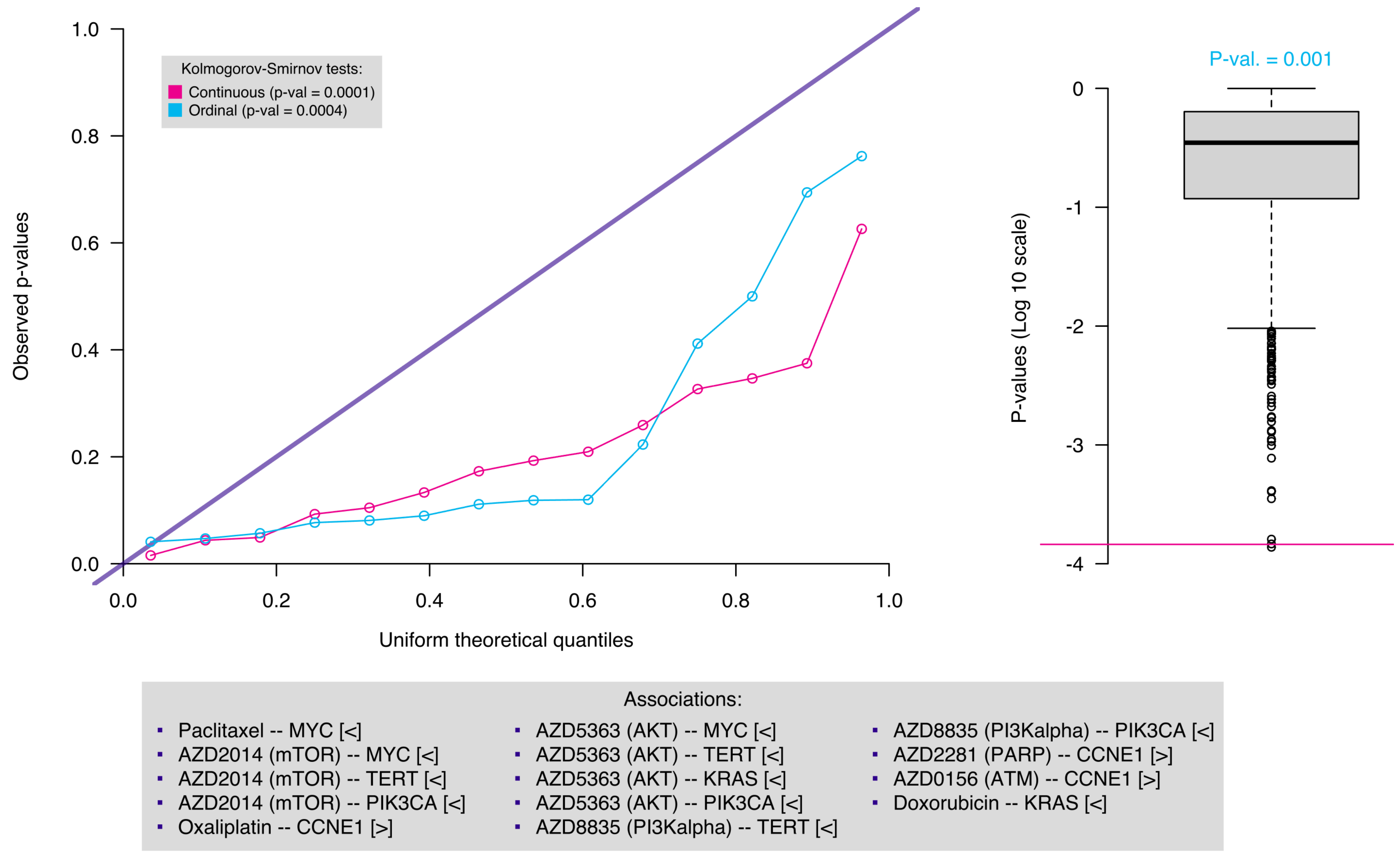

a. Left plot: Comparison of the empirical distribution functions of the p-values related to the analyses of Figures **2b**, **2d**, **2H**, **S4c** and **S4f** when considering gene copy number as a continuous (pink) or ordinal (turquoise) predictor, to the uniform distribution by means of QQ-plots. P-values of Kolmogorov–Smirnov tests (*ks.test* function of the *stats* R package) comparing the empirical distribution functions of the p-values to the uniform distribution are available. Below the plot we present the list of the *drug - gene copy number* associations of interest (n=5) with the assumed association direction. **Right plot**: boxplot of the Kolmogorov–Smirnov test p-values obtained as described above when considering 1000 random sets of *drug - gene copy number* associations. The pink line corresponds to the Kolmogorov–Smirnov test p-value obtained on the original list of pre-specified *drug - gene copy number* associations. Only one random association over a 1000 obtained a smaller p-value than the one considering the original list of pre-specified associations. **B**: the same analysis as in A with an extended set of n=14 *drug - gene copy number* associations. For boxplots in panels a and b, the central box was defined by the quantiles 0.25, 0.5 and 0.75 of the data, and the maximum whisker size equals 1.5 times of the interquartile range.

Supplementary Table 1 - Demographic characterisation of the patients with HGSOC in the OVO4 clinical cohort

		Spheroids	Tumours
**Number of Patients**		22	64
**Age**	**Median**	68	65.5
	**Range**	38-90	44-83
**FIGO Stage**	**I**		2 (3%)
	**II**	1 (5%)	1 (2%)
	**III**	9 (41%)	37 (58%)
	**IV**	12 (55%)	19 (30%)
	**Unknown**		5 (8%)
**Surgery Type**	**Primary debulking surgery**	7 (32%)	16 (25%)
	**Interval debulking surgery**	9 (41%)	44 (69%)
	**Unknown**	6 (27%)	4 (6%)
**Surgery Outcome**	**No residual**	3 (14%)	25 (39%)
	**Residual disease**	13 (59%)	24 (38%)
	**Unknown**	6 (27%)	15 (23%)
**Treatment**	**Alkylating agents (Carboplatin/Cisplatin)**	22 (100%)	63 (98%)
	**Taxanes (Paclitaxel)**	18 (82%)	56 (88%)
	**Anthracyclines (Doxorubicin/Epirubicin)**	16 (73%)	38 (59%)
	**Antimetabolites (Gemcitabine/Capecitabine)**	10 (46%)	24 (38%)
	**Anti-angiogenetic agent (Bevacizumab)**	4 (18%)	4 (6%)
	**Other**	9 (41%)	17 (27%)

Supplementary Table 2 - Characterisation of sample location, cell purity and TP53 mutation allele fraction

Patient ID	Age at Diagnosis	Tissue Site	Purity by Pathologist (%)	Purity Average (allele fraction)
22	63	omentum	40	0.1225
65	72	omentum	NA	NA
75	55	ovary	NA	NA
88	55	omentum	80	0.31
96	68	omentum	30	0.3745
101	64	omentum	NA	NA
103	70	omentum	NA	NA
124	65	omentum	NA	NA
160	73	omentum	90	0.4615
165	66	omentum	70	0.471
189	63	ovary	90	0.7245
193	74	omentum	30	NA
197	63	omentum	70	NA
203	74	omentum	70	NA
217	82	omentum	NA	NA
220	65	omentum	90	0.261
243	65	omentum	90	NA
247	75	omentum	NA	NA
261	74	omentum	20	0.191
266	62	brain met	90	0.7415
273	75	omentum	30	0.116
295	65	omentum	30	0.077
303	66	omentum	60	0.467
318	47	unknown	NA	NA
348	57	omentum	20	0.0475
357	71	omentum	60	0.209
358	68	omentum	30	0.1825
369	59	omentum	NA	NA
396	61	unknown	NA	NA
405	63	ovary nodule	30	0.091
410	76	omentum	90	0.907
413	64	omentum	70	0.7115
414	68	omentum	60	0.327
416	63	omentum	5	NA
427	69	ovary	NA	NA
434	75	ovary	30	0.3125
442	59	omentum	10	NA
461	71	ovary	NA	NA
464	75	omentum	NA	NA
466	48	omentum	80	0.407
467	44	omentum	20	0.177
469	67	omentum	40	0.1625
475	50	ovary	80	0.581
493	59	ng colon nodule	80	0.8715
495	77	omentum	NA	NA
524	70	ovary	10	0.0915
535	66	ovary left	20	0.6575
581	78	uterus	90	0.979
600	46	omentum	10	0.091
612	78	ovary	90	0.8595
629	63	ascites	90	0.684
648	58	liver metastasis	30	0.233
650	64	omentum	80	0.532
658	70	ovary	90	0.6135
716	65	omentum	80	0.454
761	83	ovary	40	0.389
771	51	ovary	90	NA
781	54	ovary	50	0.234
788	64	omentum	60	0.573
853	57	ovary	90	0.8135
855	69	omentum	NA	NA
875	70	ovary	80	0.72
891	69	ovary	70	0.4855
977	76	omentum	20	0.222
